# Proposal of metagenomic-origin LRA-5 as a precursor of active β-lactamases through Tyr69Gln and Val166Glu amino acid substitutions: a functional and structural analysis

**DOI:** 10.1128/aac.00675-25

**Published:** 2025-11-28

**Authors:** Gabriela D'Amico González, María Margarita Rodríguez, Pedro Penzotti, Florencia Brunetti, Barbara Ghiglione, Luke A. Moe, Daniela Centrón, Gabriel Gutkind, Lin Gao, Shozeb Haider, Rachel A. Powers, Sebastián Klinke, Pablo Power

**Affiliations:** 1 Facultad de Farmacia y Bioquímica, Universidad de Buenos Aires, Instituto de Investigaciones en Bacteriología y Virología Molecular (IBaViM) 28196, Buenos Aires, Argentina; 2 Consejo Nacional de Investigaciones Científicas y Técnicas (CONICET) 62873, Buenos Aires, Argentina; 3 University of Kentucky, Plant and Soil Sciences 4530, Lexington, Kentucky, USA; 4 Facultad de Medicina, Instituto de Investigaciones en Microbiología y Parasitología Médica (IMPaM), Universidad de Buenos Aires 28196, Buenos Aires, Argentina; 5 UCL School of Pharmacy 371646 , London, United Kingdom; 6 UCL Centre for Advanced Research Computing 4919 , London, United Kingdom; 7 University of Tabuk 125900, Tabuk, Saudi Arabia; 8 Department of Chemistry, Grand Valley State University 1142, Allendale, Michigan, USA; 9 Fundación Instituto Leloir, IIBBA-CONICET, and Plataforma Argentina de Biología Estructural y Metabolómica PLABEM 62898, Buenos Aires, Argentina; Universita degli studi di roma "La Sapienza", Rome, Italy

**Keywords:** Alaskan soil metagenome, X-ray crystallography, ceftazidime, β-lactamase evolution, β-lactamase precursor

## Abstract

Wild-type LRA-5, recovered from Alaskan soil samples, shares no more than 33% amino acid sequence identity with enzymes from pathogens like PER β-lactamases. Recombinant *E. coli* expressing wild-type LRA-5 and its engineered variants LRA-5^Y69Q^ and LRA-5^V166E^ showed MIC values equivalent to control strains. However, LRA-5^Y69Q/V166E^ displayed MICs above the resistant breakpoint for some β-lactams. Kinetic parameters correlated with the MICs, showing that the catalytic efficiency of LRA-5^Y69Q/V166E^ was comparable to those from class A β-lactamases, such as CTX-M-15, PER-2, and KPC-2. LRA-5^Y69Q/V166E^ exhibited *k*_cat_/*K*_m_ values up to 11,000-fold higher compared to wild-type LRA-5, which is associated with the presence of Glu166. The X-ray crystallographic structure of wild-type LRA-5 (1.80 Å; PDB 8EO5) shows that the lack of both Glu166 and a deacylation water molecule contributes to a biologically insignificant activity. Interactions observed between LRA-5 and ceftazidime (2.35 Å; PDB 8EO6) show structural conservation with other β-lactamases. In contrast, the crystallographic structure of LRA-5^Y69Q/V166E^ (2.15 Å; PDB 8EO7) bears a deacylation water molecule that is associated with the increase in catalytic activity compared to the wild-type variant. Circular dichroism results confirm that amino acid substitutions in LRA-5 do not affect the overall content of the secondary/tertiary structures. Evidence suggests that alternative evolutionary paths could have occurred for β-lactamases like LRA-5, produced by environmental microorganisms: (i) proteins having similar structural features than active β-lactamases may accumulate a small number of mutations (e.g., Y69Q/V166E) to yield active enzymes and (ii) the β-lactamase fold may have lost key residues in the absence of antibiotics.

## INTRODUCTION

The efficacy of β-lactam antibiotics has been continuously threatened by the emergence of bacterial strains resistant to virtually all available antibiotics, representing an endless challenge for the entire health system. The production of β-lactamases remains the most prevalent mechanism of resistance to β-lactam agents in Gram-negative species (1). These enzymes account today for more than 8,000 variants of the four Ambler classes (https://www.ncbi.nlm.nih.gov/pathogens/beta-lactamase-data-resources/ and http://bldb.eu) (2). The NDM-1 and KPC carbapenemases (3, 4), the “pandemic” CTX-M (5), and the “explosively” emergent carbapenem-hydrolyzing class D β-lactamases (CHDL) (6) are among the β-lactamases having more impact in clinically important pathogens.

Compelling evidence exists about the chromosomal origin of clinically important plasmid-borne β-lactamases that were likely transferred by recombination events to mobile genetic structures (i.e., transposons, plasmids, integrons) and consequently served as vehicles for dissemination of β-lactamase genes to human/animal pathogens through horizontal genetic transfer (HGT) (7, 8). Ultimately, expression levels in the hosting isolate determine its phenotypic behavior (e.g., resistant or susceptible). For example, *Kluyvera* is able to transiently colonize the human/animal gut, and antibiotic pressure in hospital settings has served as a “driving force” for the recruitment and mobilization of *bla*_CTX-M_ genes to human pathogens like *Salmonella*, *Klebsiella pneumoniae* and other enterobacteria, and even *Vibrio cholerae* (9). Other important class A β-lactamases that are suggested to have evolved from known environmental species include the PER and KPC β-lactamases, whose reservoir is most likely traced to *Pararheinheimera* and *Chromobacterium* species, respectively (10, 11). Additionally, several metallo-β-lactamases (MBLs) produced by environmental microorganisms have been identified and constitute a threat to public health if they are successfully captured and expressed in pathogens (12). This clearly depicts the important role of HGT mechanisms in the evolution of many antimicrobial resistance genes towards pathogenic species (13).

Furthermore, in the last decades, many authors have reported the risk and relevance of putative antimicrobial resistance genes in unculturable microorganisms from the resistome (14, 15). In this sense, functional metagenomics has provided access to antimicrobial resistance determinants from the environmental resistome (16–18). Antimicrobial resistance genes for various families of antibiotics from diverse environmental samples, including soil, as well as from human samples have been discovered through metagenomic analysis (19–22). Several putative β-lactamase-encoding genes have been recovered from soil sampled from the Bonanza Creek Experimental Forest near Fairbanks, Alaska. They represented all four Ambler classes, with a preponderance of MBLs. These β-lactamases were collectively named LRA (for “β-lactam resistance from Alaskan soil”). Among those found, several MBLs are related to known class B β-lactamase scaffolds from environmental microorganisms (23).

Another evolutionary mechanism that has allowed β-lactamases to gain more specialized functions is through the accumulation and selection of mutations in key amino acids that served to reshape the active site of different β-lactamases, thereby expanding their hydrolytic profile to a wider range of β-lactams and/or becoming resistant to inhibition by available inhibitors. This evolutionary pathway has been extensively documented for the extended-spectrum β-lactamases (ESBL) and inhibitor-resistant (IR) enzymes from the class A TEM and SHV β-lactamase families (24). However, none of these successful mutations occur at positions harboring strictly conserved residues that are relevant for efficient hydrolysis. Among those residues, Ser70, Lys73 (“SXXK” conserved motif), Ser130 (“SDN” motif), Lys234, Gly236 (“KTG” motif), and Glu166 (Ω loop) account for some of the invariable residues in class A β-lactamases (and their equivalent in the other serine-β-lactamases from classes C and D).

The main goal of this study was to characterize the protein encoded by the *bla*_LRA-5_ gene, a putative class A β-lactamase included in the collection of metagenomic genes from the Allen et al. study (23). Of special interest is the fact that this protein lacks glutamine and glutamic acid residues at position 69 and 166, respectively, which are important for β-lactamase function (specifically, Glu166 serves as the general base in the mechanism). Additionally, we engineered mutants where both residues were substituted with the expected ones for the known β-lactamases and assessed a likely evolutionary pathway for this enzyme. Finally, we obtained X-ray crystallographic structures for the wild-type LRA-5 (as uncomplexed enzyme and in complex with ceftazidime [CAZ]) and the variant harboring the double substitution at both the 69 and 166 positions, which allowed for a detailed description of the active site architecture.

## RESULTS AND DISCUSSION

### LRA-5 is distantly related to clinically important class A β-lactamases and lacks important residues for an active β-lactamase

As shown in Table 1, the predicted amino acid sequence of the mature LRA-5 polypeptide has nearly 68% amino acid identity with putative class A β-lactamases from the family *Pyrinomonadaceae*, which includes a single genus and species, *Pyrinomonas methylaliphatogenes*, a Gram-negative rod associated with volcanic habitats (25). Compared to other β-lactamase sequences available in databases, mature LRA-5 shares only 34%–38% amino acid identity with putative β-lactamases from other environmental microorganisms such as *Pedobacter antarcticus, Gloeobacter violaceus*, *Fischerella* sp., among others, and no more than 33% with class A β-lactamases from pathogens, like the PER ESBLs; lower identities were observed when compared to other β-lactamases like CTX-M-14 (29%), VEB-1 (32%), and PenA from *Burkholderia multivorans* (31%) (Table 1).

**TABLE 1:** Amino acid identity between LRA-5 and different class A β-lactamases

	Amino acid identity (%)						
	*Pyrinomonas methylaliphatogenes* class A	*Pedobacter antarcticus* class A	PenA	PER-1	PER-2	VEB-1	CTX-M-14
LRA-5	67.99	34.18	31.01	33.33	32.61	31.73	29.10
*P. methylaliphatogenes* class A		34.30	31.60	32.48	33.60	29.30	30.04
*P. antarcticus* class A			24.80	43.54	43.85	45.99	26.02
PenA				25.65	30.29	24.12	58.85
PER-1					88.34	40.22	24.63
PER-2						40.94	26.87
VEB-1							26.02

**Fig 1:**
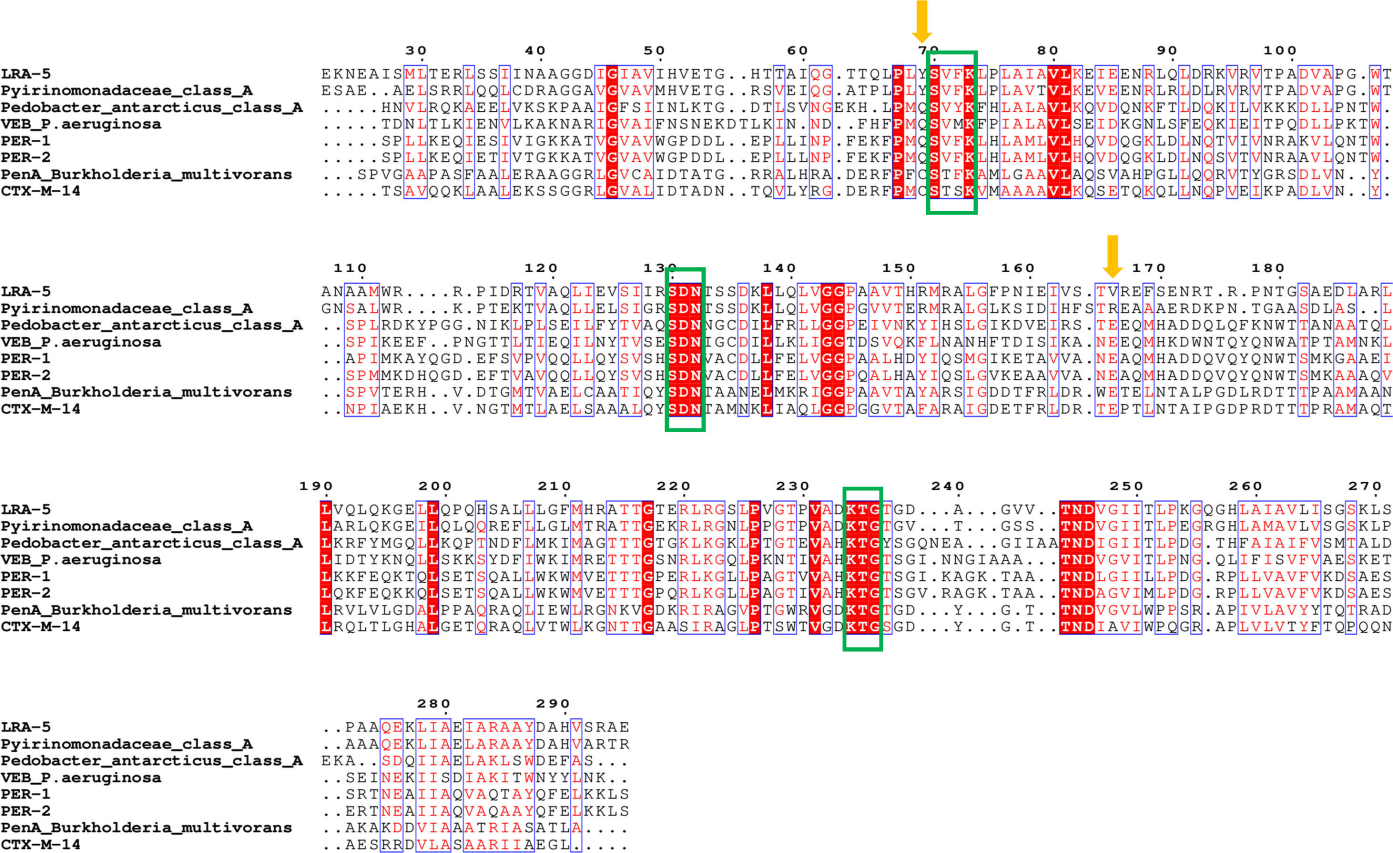
Multiple alignment of amino acid sequences of LRA-5 and other related class A β-lactamases, using the standard numbering scheme by Ambler (27). Green boxes indicate the location of the conserved motifs (SXXK, SDN, and KTG), and orange arrows indicate the location of the residues at positions 69 and 166 (tyrosine and valine in wild-type LRA-5, respectively). Residues highlighted in red background correspond to identities, whereas blue boxes indicate locations with high degree of conservation.

A sequence alignment (Fig. 1) reveals that LRA-5 bears most of the typical signatures of the class A β-lactamases: (i) the “SXXK” motif (Ser70-Val71-Phe72-Lys73) that includes the putative active site serine residue responsible for the nucleophilic attack on the β-lactam ring, (ii) the “SDN” motif (Ser130-Asp131-Asn132), and (iii) the “KTG” motif (Lys234-Thr235-Gly236). However, the sequence of LRA-5 lacks an expected glutamic acid residue at position 166, which is necessary for deacylation and release of the inactive β-lactam from the active site. In LRA-5, a valine is observed at this position; in the putative β-lactamase from *Pyrinomonadaceae*, the Glu166 residue is replaced by arginine. Another difference is the absence of glutamine, as seen for example in PER and VEB, or cysteine (PenA, CTX-M) at position 69, which is replaced by a tyrosine residue in LRA-5. Considering that Glu166 is relevant for the deacylation step in the mechanism of action (26), we anticipated that LRA-5 would have impaired activity on β-lactam substrates.

### The double substitution Tyr69Gln/Val166Glu enables hydrolytic activity against some β-lactam substrates

Allen et al. have previously demonstrated that the wild-type LRA-5, expressed from a pCC1FOS fosmid, rendered MIC values of 32 µg/mL for antibiotics like cephalexin, ceftazidime, and carbenicillin, which lie within the “resistant” range, 16 µg/mL for ampicillin and cefoxitin, and 8 µg/mL for piperacillin and amoxicillin (23).

We determined the minimum inhibitory concentrations (MICs) in *Escherichia coli* Top10 transformants harboring the *bla*_LRA-5_ alleles cloned in pUC57-Kan plasmids (Table 2). Recombinant *E. coli* producing either the wild-type LRA-5 or the different variants harboring the single substitutions, LRA-5^Y69Q^ and LRA-5^V166E^, did not show an increase of the MIC beyond the values for the recipient cells lacking the plasmids or harboring the empty vector pUC57-Kan and remained within the “susceptible” range according to the CLSI breakpoints. However, the combination of both mutations in the *bla*_LRA-5_ gene, resulting in the expression of the LRA-5^Y69Q/V166E^ variant, resulted in a phenotype characterized by MIC values above the resistant breakpoint for penicillins (not reverted by sulbactam), and cephalosporins including both oxyimino-cephalosporins ceftazidime and ceftriaxone; only aztreonam, cefoxitin, and meropenem MICs remained within the susceptible range. These results suggest that substitution of both residues at positions 69 and 166 is necessary to enable LRA-5 to acquire a β-lactamase behavior that is compatible with some of the phylogenetically closest variants like PER, VEB, and PenA β-lactamases (see Table 1).

**TABLE 2:** Minimum inhibitory concentrations (MIC) for *E. coli* Top10 transformant cells expressing the different LRA-5 variants (µg/mL)

Antibiotic	*E. coli* Top10	*E. coli* Top10 + pUC57-Kan^*b*^ harboring:				
	-	- ^*a*^	*bla*_LRA-5_	*bla*_LRA-5_^Y69Q^	*bla*_LRA-5_^V166E^	*bla*_LRA-5_^Y69Q/V166E^
Ampicillin	4	4	4	4	4	>128
Ampicillin/sulbactam	4:2	4:2	1:0.5	2:1	2:1	64:32
Cephalothin	8	8	4	8	4	32
Cefoxitin	0.5	4	0.5	1	0.5	2
Ceftazidime	0.25	0.25	0.25	0.25	0.5	16
Ceftriaxone	0.25	0.25	≤ 0.032	≤ 0.032	≤ 0.032	32
Meropenem	0.032	0.032	≤ 0.032	≤ 0.032	≤ 0.032	0.032
Aztreonam	0.25	0.25	≤ 0.125	≤ 0.125	≤ 0.125	2

^*a*^ The pUC57-Kan harbors a kanamycin resistance allele for selection.

^*b*^ Empty vector.

The kinetic parameters of wild-type LRA-5 and its double substituted variant LRA-5^Y69Q/V166E^ are shown in Table 3. As observed, the wild-type enzyme presented very low catalytic efficiency values (*k*_cat_/*K*_m_), with biologically irrelevant turnover values (*k*_cat_), below 0.05 s^−1^, despite presenting *K*_m_ values (related to the affinity of the enzyme for the substrate) comparable to other active β-lactamases (see below). This behavior is expected considering that this variant lacks Glu166, which is essential to orient the deacylation water molecule during the regeneration stage of the active site. The exceptions were nitrocefin and, strikingly, cefepime. This behavior deserves more in-depth analysis. Nevertheless, the possibility of non-enzymatic cefepime hydrolysis was disregarded as control reactions lacking LRA enzyme showed absence of unspecific degradation of the antibiotic (Fig. S1). The wild-type LRA-5 variant did not demonstrate hydrolytic activity against carbapenems or aztreonam (in this case, only marginal activity could be determined by direct hydrolysis with pure enzyme).

**TABLE 3:** Kinetic parameters of wild-type LRA-5 and its LRA-5^Y69Q/V166E^ variant^*b*^^,*c*^

	LRA-5 (wild-type)			LRA-5^Y69Q/V166E^			HEF^*a*^
Antibiotic	*K*_m_ (µM)	*k*_cat_ (s^−1^)	*k*_cat_/*K*_m_ (M^−1^ s^−1^)	*K*_m_ (µM)	*k*_cat_ (s^−1^)	*k*_cat_/*K*_m_ (M^−1^ s^−1^)	
Ampicillin	65 ± 13	0.010 ± 0.002	154 ± 44	4.0 ± 0.2	0.0020 ± 0.0006	500 ± 152	3.25
Nitrocefin	8 ± 2	0.045 ± 0.001	5,600 ± 1,405	32 ± 4	4.8 ± 0.3	150,000 ± 21,000	26.8
Cephalothin	35 ± 4	0.0050 ± 0.0008	143 ± 28	14 ± 3	0.025 ± 0.001	1,785 ± 389	12.5
Ceftazidime	6 ± 1	(5.0 ± 0.3) × 10^−5^	8.3 ± 1.5	86 ± 9	0.043 ± 0.005	500 ± 78	60.2
Ceftriaxone	11 ± 2	(3.6 ± 0.6) × 10^−6^	0.32 ± 0.08	29 ± 9	0.107 ± 0.010	3,690 ± 1,195	11,500
Cefepime	10.0 ± 0.5	0.020 ± 0.006	2,000 ± 100	108 ± 12	0.40 ± 0.02	3,700 ± 451	1.85
Aztreonam	>1,000	(2.0 ± 0.3) × 10^−5^	<0.02	19.0 ± 0.2	(1.4 ± 0.2) × 10^−4^	7.4 ± 1.1	≥370
Imipenem	ND	NH	ND	3.0 ± 0.1	0.0060 ± 0.0002	1,917 ± 86	ND
Meropenem	ND	NH	ND	5.0 ± 0.6	0.0030 ± 0.0007	600 ± 157	ND
Inhibitor	*K*_i_ (µM)	*k*_inact_ (s^−1^)	*k*_inact_/*K* (M^−1^ s^−1^)	*K*_i_ (µM)	*k*_inact_ (s^−1^)	*k*_inact_/*K* (M^−1^ s^−1^)	IEF^*a*^
Clavulanate	6.0 ± 0.6	0.0050 ± 0.0001	833 ± 102	73 ± 15	0.037 ± 0.008	507 ± 152	0.61

^*a*^ HEF, hydrolytic efficiency factor; IEF, inhibition efficiency factor.

^*b*^ ND, not determined.

^*c*^ NH, no hydrolysis was observed.

In contrast, the LRA-5^Y69Q/V166E^ variant displayed significantly higher *k*_cat_/*K*_m_ values for most of the substrates tested, compared to the variant lacking Gln69 and especially Glu166. The most affected substrates were cephalosporins, except cefepime and aztreonam, and, to a lesser extent, ampicillin. The high catalytic efficiency on ceftriaxone stands out, with a hydrolytic efficiency factor (HEF) of more than 11,000 times higher compared to the wild-type variant, and ~60× higher for ceftazidime. Aztreonam, for which the wild-type LRA-5 is estimated to have *K*_m_ values with no biological relevance (at least in the millimolar range) and negligible *k*_cat_, also showed significant improvement in the kinetic parameters in the double mutant variant. Other substrates that were hydrolyzed much more efficiently in relation to wild-type LRA-5 were nitrocefin (27×) and cephalothin (12.5×) and, to a lesser extent, ampicillin (3.25×) and cefepime (1.85×). Notably, the double- substituted variant showed a higher catalytic efficiency for imipenem compared to meropenem, the latter being similar to ceftazidime. In general, the jump in the *k*_cat_/*K*_m_ values was due to increases of up to 30,000 times (as in the case of ceftriaxone) in the turnover values (*k*_cat_), corresponding to the acquisition of Glu166 that positions the deacylation water molecule and, therefore, favors the regeneration of the active site (26, 28).

Regarding inhibition by clavulanate, both wild-type LRA-5 and LRA-5^Y69Q/V166E^ demonstrated being inhibited by clavulanate, with higher *k*_inact_ values for the LRA-5^Y69Q/V166E^ variant (7.4×), although with lower affinity, which resulted in slightly favorable inhibitory efficiency for the wild-type protein (Table 3). This behavior is not entirely striking, as it may be related to the fact that the absence of Glu166 in the wild-type variant could still favor the inhibition of clavulanate, due to the absence of the water molecule that normally “competes” with inhibitors during acylation (29).

Preliminary kinetics evaluation of individual mutants, LRA-5^Y69Q^ and LRA-5^V166E^, showed that the hydrolytic activity against β-lactams was not improved compared to the wild-type LRA-5 (data not shown). This observation evidences that the absence of both Gln69 and Glu166 seems to be a determinant for the correct functionality of the enzyme as a β-lactamase according to what was anticipated from the predictive structural models and the antibiotic susceptibility profiles. It has been well documented that the glutamate residue at position 166 is indispensable for the hydrolytic activity that characterizes serine-β-lactamases (30). In addition, it is important to note that the residue at position 166 is part of the Ω loop of class A serine β-lactamases, and its mutation to a different residue could also give rise to structural disorder in that domain that is unfavorable for the correct folding of the loop, resulting in suboptimal enzymatic activity (see below).

We also compared the catalytic efficiencies of the LRA-5 variant carrying the double substitution with other relevant class A β-lactamases found in clinically important microorganisms, such as CTX-M-15, PER-2, and KPC-2 (Table 4). Although LRA-5 has, indeed, acquired hydrolytic activity against several β-lactams in comparison to the wild-type variant, the *k*_cat_*/K*_m_ values, in general, are still several orders of magnitude lower than for the other class A enzymes. PER-2 and CTX-M-15 display higher catalytic efficiencies for the oxyimino-cephalosporins although the behavior of LRA-5^Y69Q/V166E^ against ceftazidime and cefepime is comparable to CTX-M-15 and KPC-2, respectively.

**TABLE 4:** Comparative catalytic efficiencies of LRA-5^Y69Q/V166E^ and other clinically important class A β-lactamases

	*k*_cat_/*K*_m_ (M^−1^s^−1^)			
	LRA-5^Y69Q/V166E^	PER-2 (31)	CTX-M-15 (32)	KPC-2 (33)
Ampicillin^*a*^	500	3,000,000	500,000	500,000
Cephalothin	1,785	3,900,000	500,000	8,300,000
Ceftriaxone^*b*^	3,690	1,800,000	3,500,000	350,000
Ceftazidime	500	710,000	1,000	15,000
Cefepime	3,700	470,000	10,000	6,700
Aztreonam	7.4	400,000	100,000	300,000
Imipenem	1,917	16,000	ND^*c*^	1,000,000

^*a*^ Ampicillin or amoxicillin.

^*b*^ Ceftriaxone or cefotaxime.

^*c*^ ND, not determined.

Even if the kinetic parameters obtained cannot be strictly correlated with those of MICs, this could be explained by the fact that the expression of the *bla*_LRA_ genes for the MIC determination was achieved from constructions in a high-copy number vector, which might not reflect the actual scenario in their putative natural host, where those genes are likely expressed from the core chromosome and therefore in lower levels. Nevertheless, these results serve to demonstrate the effect of the double amino acid substitution in the phenotypic and kinetic behavior of LRA-5.

### Structural analysis of wild-type LRA-5 and LRA-5^Y69Q/V166E^

All variants of LRA-5 crystallized in space group *P*2_1_2_1_2, and crystals diffracted at a final resolution of 1.80 Å (uncomplexed LRA-5; PDB 8EO5), 2.35 Å (LRA-5/ceftazidime; PDB 8EO6), and 2.15 Å (LRA-5^Y69Q/V166E^; PDB 8EO7). Main crystallographic data and refinement statistics are shown in Table 5. All structures contain two monomers per asymmetric unit (chains A and B). Due to the presence of two additional residues in relation to other enzymes, and to maintain the consensus Ambler nomenclature for class A β-lactamases (27), they were numbered as Pro103A (between Ala103 and Gly104) and Ala108A (between Asn108 and Ala109); in addition, the positions 175 and 176 are skipped in the numbering.

**TABLE 5:** X-ray data collection and refinement statistics

	LRA-5	LRA-5/CAZ	LRA-5^Y69Q/V166E^
Data collection:			
Synchrotron source	SOLEIL	SOLEIL	DIAMOND
Beamline	PROXIMA-2A	PROXIMA-2A	I03
Detector	EIGER X 9M	EIGER X 9M	EIGER2 XE 16M
Crystal-detector distance (mm)	176.21	175.76	288.70
Rotation range/image (°)	0.1	0.1	0.1
No. of frames	3,600	3,600	3,600
Exposure time/image (s)	0.0043	0.0043	0.0080
Wavelength (Å)	0.9801	0.9801	0.9763
Space group	*P*2_1_2_1_2	*P*2_1_2_1_2	*P*2_1_2_1_2
Unit cell parameters			
*a*, *b*, *c* (Å)	74.27, 162.62, 71.00	74.08, 162.11, 70.27	73.86, 162.24, 70.28
*α*, *β*, *γ* (°)	90, 90, 90	90, 90, 90	90, 90, 90
Resolution range (Å)*^a^*	48.94–1.80 (1.91–1.80)	43.66–2.35 (2.49–2.35)	48.58–2.15 (2.22–2.15)
Total reflections	1,088,788 (176,136)	486,529 (71,922)	632,737 (55,824)
Unique reflections	80,573 (12,790)	36,200 (5,654)	46,812 (4,010)
Redundancy	13.5 (13.8)	13.4 (12.7)	13.5 (13.9)
Completeness (%)	99.9 (99.5)	99.7 (98.1)	100.0 (100.0)
Mean I/σ(I)	15.6 (1.4)	11.8 (1.6)	7.3 (1.1)
Overall Wilson *B*-factor (Å^2^)	30	41	40
*R*_meas_	0.131 (2.172)	0.283 (2.330)	0.260 (2.823)
*R*_pim_	0.039 (0.713)	0.096 (0.731)	0.070 (0.750)
CC_(1/2)_	0.999 (0.556)	0.997 (0.495)	0.997 (0.687)
Subunits/asymmetric unit	2	2	2
Refinement:			
Reflections used in refinement	80,557	36,122	43,641
*R*_work_	0.189	0.196	0.225
*R*_free_	0.215	0.216	0.248
No. of non-hydrogen atoms			
All atoms	4,322	4,120	3,858
Macromolecules	4,043	4,016	3,756
Ligands	8	62	–^*c*^
Solvent	271	42	102
RMS*^b^* deviations from ideal values			
Bonds (Å)	0.017	0.006	0.005
Angles (°)	1.47	1.04	0.96
Average *B*-factor (Å^2^)			
All atoms	37	46	48
Protein	37	46	48
Ligand	41	59	–^*c*^
Solvent	40	41	48
Ramachandran plot			
Favored regions (%)	97.9	98.1	98.0
Allowed regions (%)	2.1	1.9	2.0
Outliers (%)	0	0	0
Rotamer outliers (%)	0	2.1	0
Deposition:			
PDB code	8EO5	8EO6	8EO7

^*a*^ Statistics for the highest resolution shell are given in parentheses.

^*b*^ RMS, Root-mean square.

^*c*^ –, no ligand is present.

The protein chain for the uncomplexed-enzyme (ligand-free) LRA-5 includes 270 residues (Lys23-Ser292) in the A chain and 268 (Ala26-Arg293) in the B chain. The LRA-5/CAZ complex contains 268 residues (Glu25-Ser292) in both chains. Finally, the LRA-5^Y69Q/V166E^ structure includes 255 residues in the A chain (Glu25-Val102, Met110-Ser170, and Arg177-Ser292) and 248 in the B chain (Ala26-Ala100, Pro114-Ser170, and Arg177-Ser292). The structures are solvated by 271 (uncomplexed LRA-5), 42 (LRA-5/CAZ complex), and 102 (double mutant LRA-5) ordered water molecules. The electron density map is well defined along the main chain of both monomers in uncomplexed-LRA-5 and in its complex with CAZ; in the structure of LRA-5^Y69Q/V166E^, the regions between residues 103–109 and 171–176 (chain A) and 101–113 and 171–176 (chain B) were not well defined and were, therefore, excluded from the final model.

The average values of root mean square deviations (rmsd) for C^α^ atoms between the main chains of LRA-5 and its complex with CAZ is 0.31 Å, while between wild-type LRA-5 and LRA-5^Y69Q/V166E^, it is 0.28 Å. These low values indicate that both the binding of a ligand and the occurrence of two substitutions do not seem to affect the overall folding of the protein. Therefore, unless otherwise noted, the following description and discussion will be based on monomer A and considered equivalent to monomer B.

**Fig 2:**
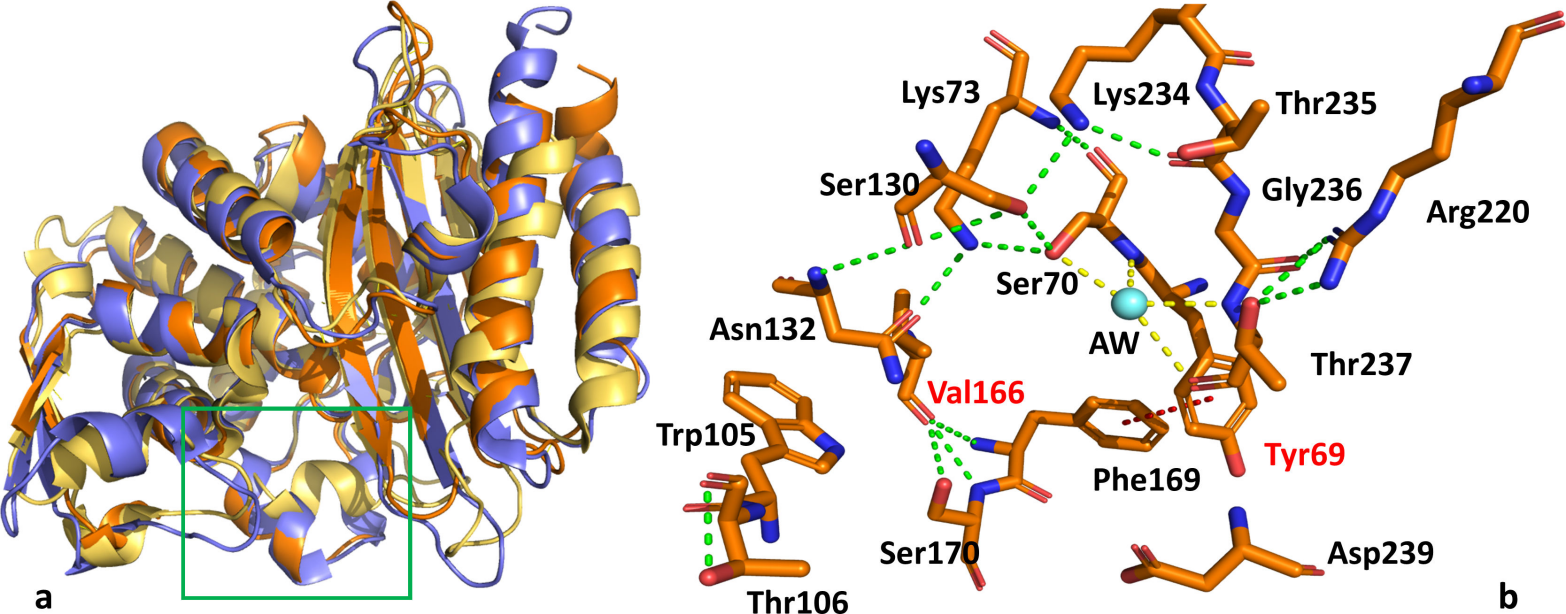
(**a**) Superposed view of LRA-5 (PDB 8EO5; orange), PER-2 (PDB 4D2O; blue), and CTX-M-14 (PDB 6CYK; yellow). The green box indicates the location of the Ω loops (**b**) Architecture of the active site of wild-type LRA-5. References: cyan sphere: acylation water molecule (AW); green dashed lines: hydrogen bonds; red dashed line: π-π stacking interaction. Residues that differ between the active β-lactamases are shown in red labels. This and all subsequent figures were created with PyMOL (34).

The structure of the uncomplexed form of LRA-5 shows an overall organization similar to class A β-lactamases (Fig. 2a), preserving the “α/β” and “α domains,” and with the catalytic cleft at the interface between both domains. In addition, despite the lack of Glu166, its Ω loop seems to share the unique structural folding with other β-lactamases like the PER variants.

The putative active site of LRA-5 in its uncomplexed form (PDB 8EO5) includes the most important amino acid residues located in conserved positions in class A β-lactamases (Fig. 2b), which create a hydrogen bond (HB) coordination network for the stabilization of the catalytic pocket. Thus, HBs are observed between residues important for catalysis (i.e., Ser70, Lys73, Ser130), residues that participate in guiding the positioning of substrates within the active site (i.e., Asn132, Lys234, Thr235, Thr237), other amino acids like Arg220 that can participate through second sphere coordination interactions, and a water molecule located in an equivalent position to the acylation water molecule (AW) in other structures. In addition, unusual features are observed, compared to other serine-β-lactamases: (i) Val166 (which is replaced by Glu in active enzymes) interacts *via* HB through its carbonyl group with Ser170 and Phe169, and its side chain does not point to the “core” of the enzyme; (ii) a deacylation water (DAW) is not present, likely due to the absence of a glutamic acid residue, which is normally essential for stabilizing that water molecule; (iii) Tyr69 (which in some enzymes is replaced by Gln) apparently forms a “π-π” type interaction with the aromatic ring of Phe169, which is also unusual in other serine-β-lactamases. This latter hydrophobic interaction could contribute, together with the absence of Glu166, to the biologically insignificant activity of the wild-type LRA-5 variant against the antibiotics tested, likely due to the larger size and intrinsic hydrophobic nature of tyrosine (compared to glutamine) and clashes created by its position at the entrance of the active site cavity. As mentioned above, the deacylation water molecule is not present since enzymes lacking Glu166 would not be able to orient that catalytic water molecule for the final stage of the catalytic mechanism of an active β-lactamase, namely, the regeneration of the active site and release of the hydrolyzed and inactive antibiotic molecule (28, 35).

**Fig 3:**
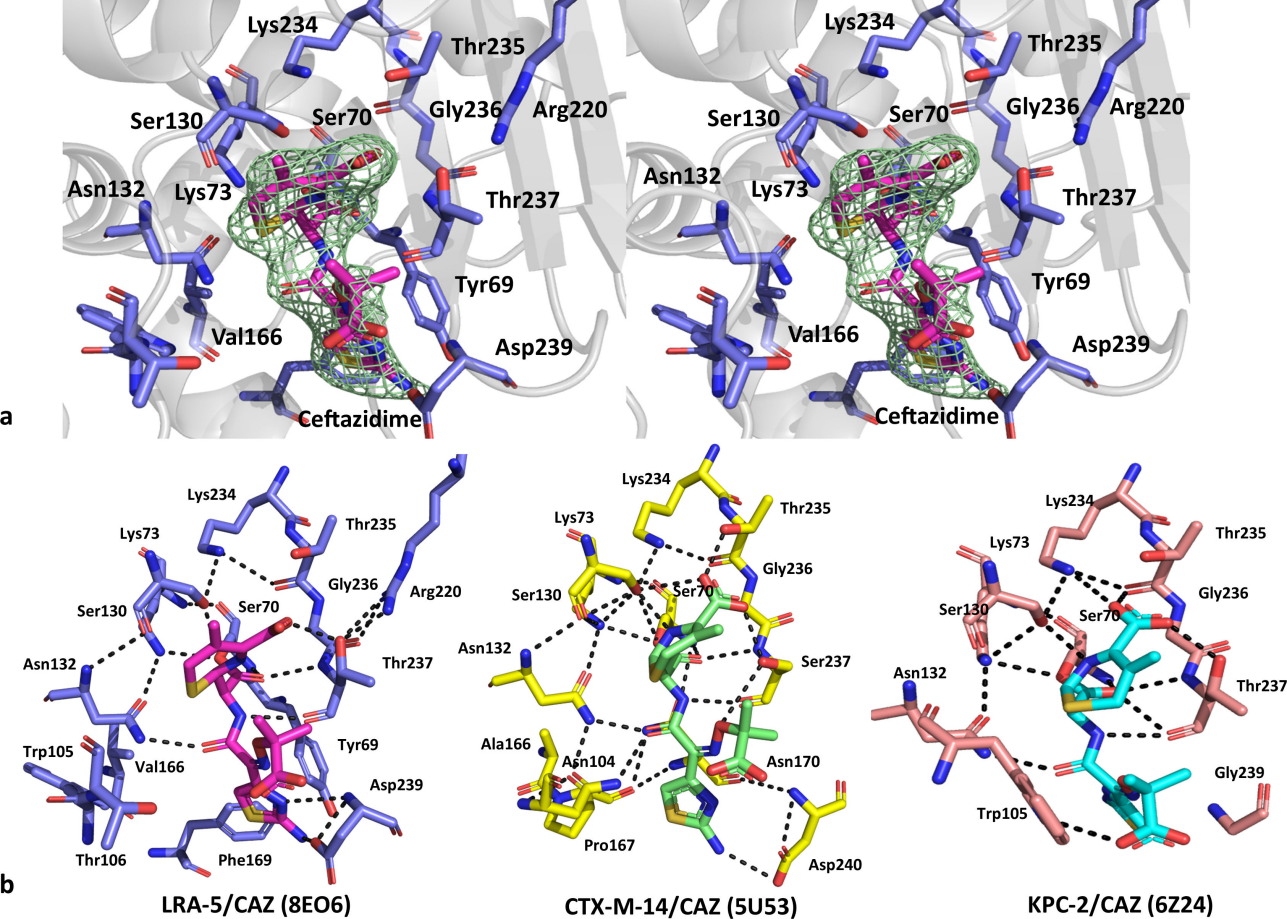
(**a**) Detail of the interaction of ceftazidime (magenta sticks) in the active site of LRA-5, showing the Polder omit map (green mesh) contoured at 4 σ around the ligand (stereo view). (**b**) Comparative view of the interaction of ceftazidime with LRA-5 (blue; PDB 8EO6), CTX-M-14 (yellow; PDB 5U53), and KPC-2 (salmon; PDB 6Z24). Main hydrogen bonds are shown in black dashed lines.

The structure of LRA-5 in complex with ceftazidime (PDB 8EO6) shows clear and well-defined electron density for the ligand bound to Ser70 *via* a covalent linkage between the O_γ_ atom of Ser70 and the carbonyl carbon moiety of the ligand, after cleavage of the N_5_-C_8_ amide bond of the β-lactam ring (Fig. 3a). The ceftazidime molecule (CAZ) exhibits HB interactions with several LRA-5 residues: (i) the CAZ O_9_ atom is oriented toward the oxyanionic pocket of the enzyme, forming important HBs with the amide N atoms of Ser70 and Thr237; (ii) Asn132 coordinates with the O_12_ atom of CAZ by means of its N_δ2_ atom; (iii) Thr237 forms HBs between its terminal O_γ1_ atom and the C4 carboxylate group of the dihydrothiazine ring of the ligand, and between its carbonyl O atom and the N_10_ atom of CAZ, and (iv) Asp239 forms HBs not described in other β-lactamases with the terminal aminothiazole ring of CAZ. Also, the complex is stabilized by additional intramolecular HB interactions, highlighting the connection between Thr237 and Arg220, observed in other enzymes such as PER and KPC (31, 36). Other relevant observations correspond to the presence of a strong hydrophobic center between the aromatic systems of Tyr69, Phe169, the side chains of Val242 and Val243, and the aminothiazole ring of CAZ, the displacement toward the solvent of the side chain of Arg173 by the positioning of the CAZ molecule (not shown), and the absence of the acylation water molecule, whose place is occupied by the carbonyl group of the β-lactam ring, and has most likely been displaced upon binding of CAZ.

The structure of the LRA-5/CAZ complex was compared by superposition with other class A β-lactamases: CTX-M-14 (PDB 5U53) and KPC-2 (PDB 6Z24) (Fig. 3b); both structures correspond to mutants where the Glu166 residue is replaced by Ala and Gln, respectively. The average rmsd values for Cα atoms between the main chains of LRA-5/CAZ and the complexes with CTX-M-14 and KPC-2 were 1.67 and 1.75 Å, respectively. On the other hand, the rmsd values for Cα atoms within the active site were 1.16 and 0.58 Å for CTX-M-14 and KPC-2, respectively, suggesting higher conservation within the active site when CAZ is bound. The orientation of the CAZ molecule is equivalent in all structures, with the carbonyl group (C_8_-O_9_) of the hydrolyzed β-lactam ring oriented toward the oxyanionic pocket located between Ser70 and Thr237 (LRA-5, KPC-2) or Ser237 (CTX-M-14), sharing many HB interactions with conserved residues from the active site. Among the dissimilar interactions between them, the following stand out: CAZ forms HBs with Asn104 and Asp240 in CTX-M-14 and with Trp105 and Gly239 in KPC-2.

Interestingly, we obtained several complete X-ray diffraction data sets of LRA-5^Y69Q/V166E^ crystals soaked with CAZ (the best at 2.35 Å), but no ligand density was found in any of the active sites even after several attempts using the same reagents, ligand concentrations, and soaking times as for the wild-type enzyme crystals.

**Fig 4:**
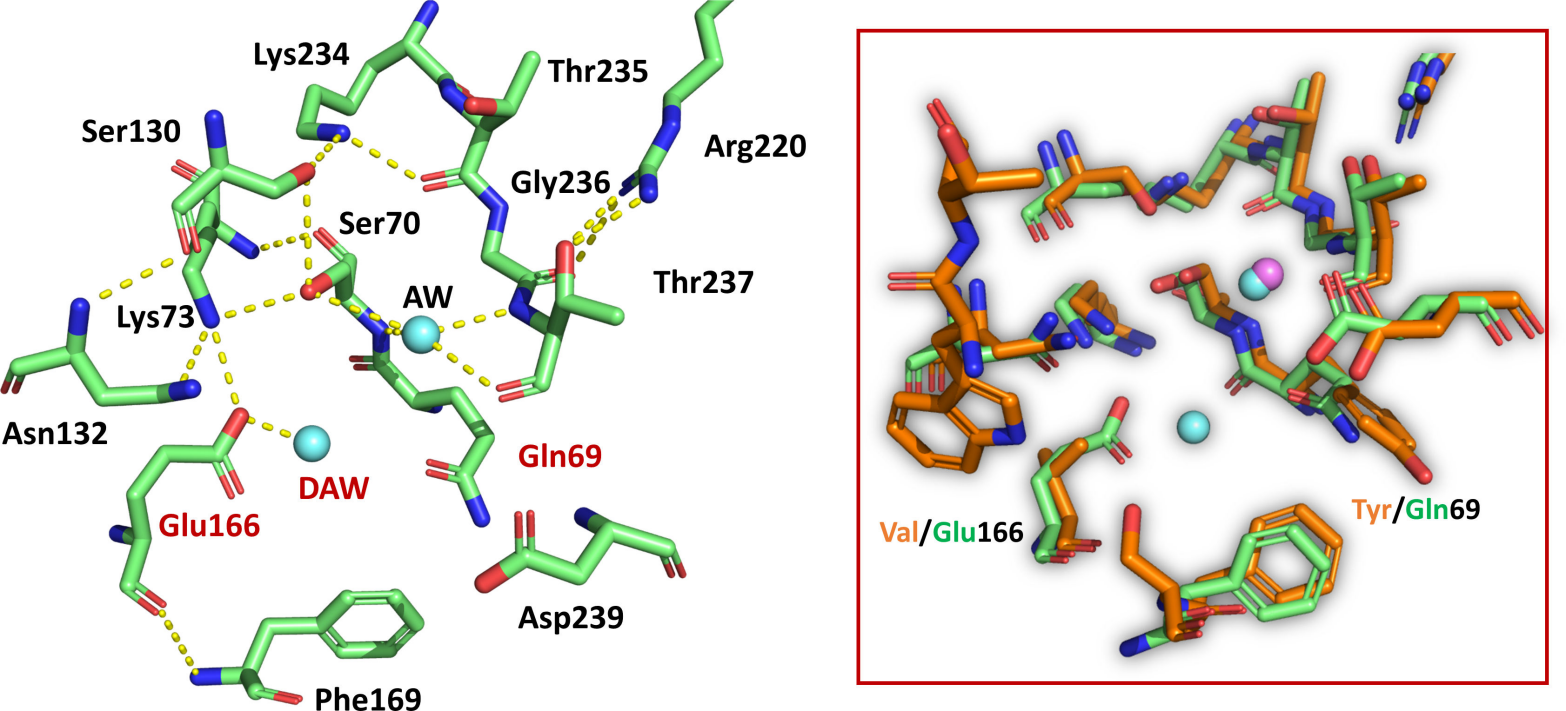
Left panel: Active site of LRA-5^Y69Q/V166E^ (PDB 8EO7). The double substitution that incorporates glutamine and glutamic acid at positions 69 and 166, respectively (red labels), restores the hydrogen bonds network present in active class A β-lactamases (yellow dashed lines), allowing the positioning of the putative deacylating water (DAW) associated with Glu166. Water molecules are depicted as light-blue spheres. Right panel: superposition of the active sites of wild-type LRA-5 and LRA-5^Y69Q/V166E^, highlighting the Tyr/Gln69 and Val/Glu166 residues, and the conserved active site water molecules; the violet sphere represents the acylating water molecule in wild-type LRA-5.

The introduction of the two mutations that generate the LRA-5^Y69Q/V166E^ variant gave rise to an enzyme with an active site architecture equivalent to that of active class A β-lactamases. In the crystallographic structure refined at 2.15 Å (PDB 8EO7), the incorporation of both catalytic water molecules is clearly observed, compared to the wild-type LRA-5 (Fig. 4). The presence of the deacylation water molecule in the LRA-5^Y69Q/V166E^ variant supports the fact that this mutational variant presents a considerable increase in catalytic activity, as discussed previously when analyzing the kinetic parameters. We hypothesize that the lack of bound CAZ in the soaked LRA-5^Y69Q/V166E^ crystals might be the consequence of the modification in the electrostatic environment mainly around position 69, where the presence of a Gln residue instead of a Tyr precludes the interactions between the aromatic systems of the latter residue with Phe169 and with the aminothiazole ring of CAZ. The lack of ligand in the crystal structure is also in line with a *K*_m_ value more than one order of magnitude higher for the double mutant in comparison with wild-type LRA-5 (Table 3).

Therefore, it would be possible for an enzyme with no or negligible hydrolytic activity against β-lactam substrates to evolve through a handful of mutations towards variants with a behavior compatible with a β-lactamase. This represents an unprecedented finding, demonstrating the possible existence of an evolutionary path from inactive enzymes to others with specific hydrolytic activity, through selected mutations in key residues.

### Impact of the amino acid substitutions in the structural conformation of LRA-5

**Fig 5:**
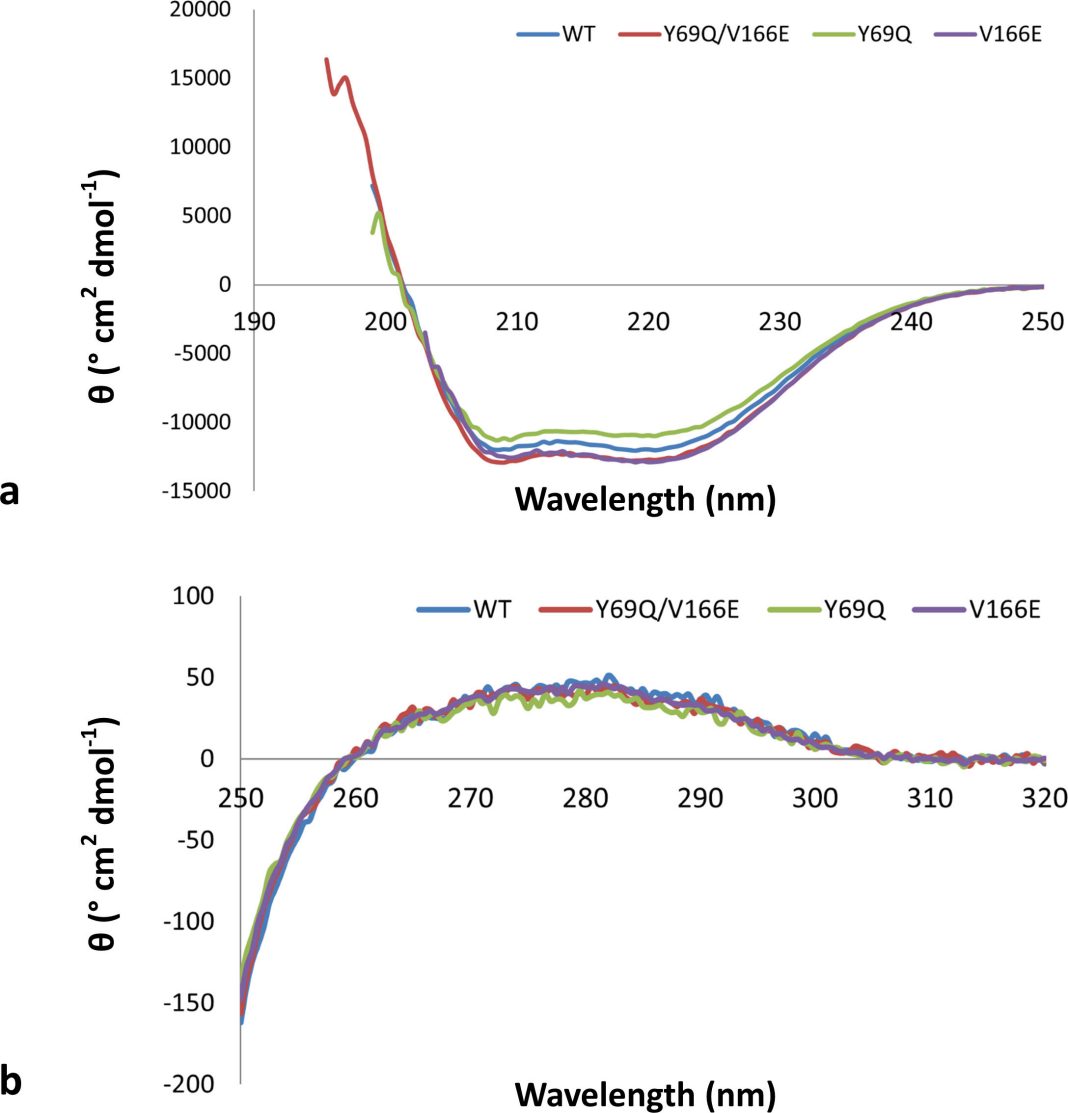
(**a**) Far and (**b**) near UV-circular dichroism (CD) spectra for LRA variants. Color codes are indicated in the plots.

Biophysical studies by circular dichroism (CD) were performed to evaluate the impact of the amino acid substitutions in the overall structure of the LRA-variants (Fig. 5). Although slight differences were observed, CD spectra indicate the preservation of both the secondary and tertiary structure for all variants of LRA-5. In line with the crystallographic findings, we can affirm that the mutational changes Tyr69Gln and Val166Glu in the LRA-5 sequence, either as single or double substitutions, do not generate substantial changes in the overall content and distribution of the secondary and tertiary structures. Therefore, considering that the enzymes have a similar folding, it is likely that the differences in activity are mainly due to the influence of the amino acid substitutions and not a perturbation in the structural stability. Other additional studies, such as thermal stability assays, could provide more conclusive evidence.

### Enhanced sampling molecular dynamics simulations

To further investigate how point mutations alter the conformational dynamics of the LRA-5 and its single and double substituted variants, we performed enhanced sampling molecular dynamics simulations and compared the structural changes. Our analysis focused on the backbone dihedral angles of the residues Tyr69 and Val166 in the wild-type protein, and Gln69 and Glu166 in the variants. These backbone torsional angles play a central role in defining the accessible conformational states as they capture some of the slowest, large-scale motions inherent to the system (37). Moreover, they are highly sensitive to amino acid substitutions and have been shown to provide a direct measure of the structural perturbations induced by mutations in β-lactamases (38). For this reason, we selected these angles as the most suitable collective variables (CVs), denoted as CV1 = *ϕ* and CV2 = *ψ*. Our simulations revealed that the system explores the conformational landscape efficiently, with the trajectories showing rapid diffusion across the full span of the CV space (Fig. S2). Over the course of the 5 μs of sampling, the systems explored all relevant free energy minima essential for describing their conformational dynamics (Fig. S3). From the well-tempered metadynamics (wt-MetaD) trajectories of the wild-type system, distinct structural states were identified based on the values of CV1 and CV2, and these were subsequently used as representative conformers to further probe the dynamic and mechanistic consequences of the mutations.

Focusing on Tyr69, both wild-type LRA-5 (CV focused on Tyr69) and the LRA-5^Y69Q^ mutant sample five free energy basins (A-E; Fig. S4). While the Tyr69 residue in the wild-type LRA-5 protein primarily populated basin D, the lowest energy minimum, the LRA-5^Y69Q^ substitution increased conformational flexibility, allowing the system to also explore basin C. In contrast, the Glu166 residue in the wild-type protein was localized within basin C (Fig. S5). Substitution of Val166 by glutamate markedly reshaped the free energy landscape where basin C disappeared, and a new minimum emerged as basin D. Basin B, which was previously only sparsely populated, became the global minimum.

Due to the high computational cost of employing more than two collective variables (CVs) in wt-MetaD, we instead employed OneOPES simulations to investigate the dynamics of the double substituted LRA-5^Y69Q/V166E^ variant. In this approach, the *ϕ* and *ψ* dihedral angles of both substituted residues (Gln69 and Glu166) were simultaneously sampled to construct the free energy landscape. As observed in the wt-MetaD simulations, the OneOPES simulations converged within the 4 μs sampling time (Fig. S6 and S7). The Gln69 backbone displayed enhanced conformational flexibility, exploring three isoenergetic free energy minima (Fig. S8). In contrast, while Val166 in the wild-type enzyme explored basins B, C, and D, the substitution by glutamate restricted Glu166 dynamics only to basins A, B, and C, with basin D disappearing entirely (Fig. S9). The differences between the wild-type and double substituted LRA-5^Y69Q/V166E^ variants highlight the differential impact of the two substitutions on the accessible conformational space.

The conformations of the wild-type (PDB 8EO5) and the double mutant LRA-5^Y69Q/V166E^ (PDB 8EO7) crystal structures match closely onto representative basin structures, with root mean square deviations (RMSD) of less than 1.0 Å (Fig. S10). In the double mutant, the overall topology and spatial positioning of Gln69 and Glu166 in the basin-derived conformations align well with those observed in the crystal structure (PDB 8EO7). In contrast, this correspondence does not extend to the side chains of Tyr69Gln and Val166Glu in the single mutants, where deviations from the crystallographic orientations are evident (Fig. S10). Based on our experimental, structural and computational data, we speculate that the side chain misalignment could be a contributing factor to the suboptimal enzymatic activity of the single substitutions.

### Conclusions

In this work, we presented a thorough functional and structural description of LRA-5, a protein that had been previously detected from metagenomes of Alaskan soils using functional metagenomics, as well as a detailed study of engineered variants harboring different amino acid substitutions.

The LRA-5 encoding gene is devoid of promoter sequences and is surrounded by genes with metabolic functions, and according to the characteristics of its sequence and initial phenotypic properties, it had been proposed as a new variant within the serine-β-lactamases. However, both its sequence and structure reveal several inconsistent structural features, namely an atypical Ω loop fold, and the absence of one of the most critical residues for β-lactamase functionality (a valine residue instead of glutamic acid at position 166), whose importance in the catalysis of β-lactams, by properly orienting the deacylation water molecule for efficient product release and regeneration of the active site, is undisputed.

We also succeeded in generating an enzyme derived from LRA-5 capable of hydrolyzing β-lactam antibiotics by incorporating only a couple *in vitro* mutations (Tyr69Gln and Val166Glu), and this variant demonstrated structural and functional features compatible with a serine-β-lactamase. Although the observed kinetic behavior denotes an enzyme with catalytic efficiencies (*k*_cat_/*K*_m_) 2–3 orders of magnitude lower than “clinically-relevant” variants, the amino acid substitutions likely provide a favorable structural environment (i.e., proper interactions with the ligand and positioning of catalytic water molecules) that renders an enzyme able to hydrolyze β-lactam antibiotics. This activity, added to other factors inherent to the producing microorganisms (e.g., high-level expression in *E. coli* transformants), could result in a clearly decreased susceptibility to β-lactams. Considering that LRA-5 was isolated from supposedly pristine environmental niches, devoid of (or with scarce) antibiotic pressure, we, therefore, hypothesize that the emergence of enzymes capable of hydrolyzing β-lactam antibiotics can occur independently from the presence of selective stimuli (i.e., antibiotics in the environment).

So far, it has been accepted that β-lactamases follow two main evolutionary paths, supported by experimental evidence: (i) mutations in genes generally harbored by mobile elements that, upon antibiotic use, selected new variants with remodeled structure enough for yielding more specialized activities, either toward last resort antibiotics or β-lactamase inhibitors; or (ii) resident genes in the chromosome of environmental species, encoding for enzymes with native extended-spectrum or even carbapenemase activity, which were recruited by mobile elements and disseminated to clinical pathogens. In both cases, the “primary” gene already encodes for a protein able to inactivate β-lactams.

Our evidence suggests that alternative evolutionary paths could have occurred for some β-lactamases. In this regard, we need to consider the possibility that proteins produced by environmental microorganisms (represented by wild-type LRA-5) with similar folding and structural features than active β-lactamases may accumulate a small number of mutations to yield proteins with hydrolytic activity towards β-lactams; LRA-5^Y69Q/V166E^ would be a representative. In the opposite direction, it is also likely that the β-lactamase fold, in the absence of antibiotics, may have lost some of its key residues. Nevertheless, these hypotheses need to be supported by additional experiments, which are beyond the aims of this article.

## MATERIALS AND METHODS

Parts of the methods were adapted from reference 32 for this study.

### Bacterial strains, plasmids, and synthetic genes

The pCC1FOS/βLR5 fosmid containing a metagenomic DNA fragment including the *bla*_LRA-5_ gene, obtained in the study by Allen et al. (23), was generously delivered by Dr. Handelsman. *E. coli* ATCC 25922 was used for quality control in antimicrobial susceptibility assays. *E. coli* Top10 (Invitrogen, USA) and *E. coli* BL21(DE3) (Novagen, USA) were hosts for transformation experiments. Plasmid vectors pGEM-T Easy Vector (Promega, USA) and kanamycin-resistant pET28a(+) (Novagen, Germany) were employed for routine cloning experiments and for β-lactamase purification, respectively. Genes encoding for the variants LRA-5^Y69Q^, LRA-5^V166E^, and LRA-5^Y69Q/V166E^ were obtained by synthesis in the pUC57-Kan vector (Gene Universal Inc, USA), for MIC determination.

### Recombinant DNA methodologies

The wild-type *bla*_LRA-5_ gene was amplified by PCR from the recombinant fosmid pβLR5, using 3 U Pfu DNA polymerase (ThermoScientific, USA) and 1 µM LRA-5-NdeF2 (5′ TCGGGCCATATGTACCAAAGCTCTC-3′) and LRA-5-XhoR2 (5′- CTCCTTTCTCGAGTGCTCACTCCG-3′) primers, containing the *Nde*I and *Xho*I restriction sites, respectively (underlined in the sequences). The PCR product was first ligated in a pGEM-T vector and introduced in *E. coli* Top10 competent cells by transformation, and the insert was sequenced for verification of the identity of *bla*_LRA-5_. The resulting recombinant plasmid was then digested with *Nde*I and *Xho*I, and the released insert was purified and cloned in the corresponding *Nde*I-*Xho*I sites of a pET28a(+) vector. The ligation mixture was used to transform *E. coli* BL21(DE3) competent cells and cultured on LBA plates supplemented with 30 µg/mL kanamycin. Selected positive recombinant clones were sequenced to confirm the identity of the *bla*_LRA-5_ gene, and the recombinant *E. coli* clone harboring the pET28/LRA-5 plasmid was obtained for protein expression experiments, named *E. coli* BL21/LRA-5. DNA sequences were determined at Macrogen Inc. (South Korea). Nucleotide and amino acid sequence analyses were performed with the NCBI (http://www.ncbi.nlm.nih.gov/) and ExPASy (http://www.expasy.org/) analysis tools.

### Antimicrobial susceptibility

Plasmids harboring synthetic genes (pUC57-Kan based constructions) were introduced by transformation in *E. coli* Top10 cells. Resulting transformant cells were used for the determination of the minimum inhibitory concentrations (MICs) of β-lactam antibiotics by the broth microdilution method in 96-well microtiter plates incubated 18 h at 35°C, following the CLSI’s original guidelines (39).

### Production and purification of LRA-5 variants

Overnight cultures of recombinant *E. coli* BL21(DE3) cells expressing wild-type LRA-5 and LRA-5^Y69Q/V166E^ were diluted (1/50) in 500 mL Lysogeny Broth (LB) supplemented with 30 µg/mL kanamycin and grown at 37°C until 0.6–0.8 OD units (*λ* = 600 nm) was reached. At this point, 1 mM isopropyl β-D-thiogalactopyranoside (IPTG) was added, and cultures were resumed for growing overnight at 20°C (200 rpm stirring). Then, cell pellets were obtained after centrifugation at 8,000 rpm for 20 min (4°C) in a Sorvall RC-5C (GS3 rotor) and resuspended in 20 mM Tris buffer (pH 7.5) + 500 mM NaCl (buffer A). Crude extracts were obtained by ultrasonic disruption (9 cycles of 5 min, 40% potency, 20 pulses/min, on ice bath) and clarified by centrifugation at 10,000 rpm for 20 min (4°C, SS34 rotor). Clarified supernatants were filtrated through 0.45 µm pore-size membranes, and loaded onto 5 mL HisTrap HP affinity columns (GE Healthcare Life Sciences, USA), connected to an ÄKTA-purifier (GE Healthcare, Uppsala, Sweden), and equilibrated with buffer A. The column was extensively washed to remove unbound proteins, and the LRA-5 proteins were eluted using a linear gradient (0%–100%; 1 mL/min flow rate) of buffer B consisting of buffer A + 500 mM imidazole (pH 7.5). Eluted fractions were analyzed by SDS-PAGE in 15% polyacrylamide gels. Fractions containing the pure LRA-5 variants were dialyzed against buffer A, and the histidine tag was removed by thrombin digestion (16 h at 25°C), using 10 U thrombin per mg protein for complete proteolysis, and later by affinity purification using 1 mL HisTrap HP columns (GE Healthcare Life Sciences, USA), following the same conditions as before (the pure LRA-5 is recovered in the pass-through, while the His-tag and any uncleaved fractions remain attached to the column). The protein purity was assessed by Coomassie blue staining on 15% SDS polyacrylamide gels, and protein concentration was determined by UV absorbance at 280 nm and calculated according to the Lambert-Beer law. The fractions of purified enzymes were stored at −80°C for subsequent kinetics and crystallographic assays.

### Steady-state enzyme kinetics

Steady-state kinetic parameters were determined using a T80 UV/VIS spectrophotometer (PG Instruments Ltd, UK), monitoring the hydrolysis of β-lactams substrates by pure enzymes as the absorbance variation within 5–10 min, at the corresponding wavelength. Briefly, each assay was completed in 20 mM Tris buffer (pH 7.5), in a total volume of 500 µL at room temperature (22°C). The steady-state kinetic parameters *K*_m_ and *V*_max_ were obtained under initial-rate as described previously (40), with non-linear least squares fit of the data to fulfill the Henri Michaelis-Menten model, according to equation 1:


(1) $$v=\frac{V_{max}\times [S]}{K_{m}+[S]}$$

For low *K*_m_ values, the *k*_cat_ values were derived by complete hydrolysis time courses as described by De Meester et al. (41). For poor substrates that behave as competitive inhibitors, the *K_i_* was obtained by monitoring the residual activity of the enzyme in the presence of various concentrations of the drug and 100 µM nitrocefin as reporter substrate; the corrected *K*_i_ (considered the observed or apparent *K*_m_) value was finally determined using equation 2:


(2) $$K_{i}\;=\;\frac{K_{i\;obs}}{\frac{\left(1+\left[S\right]\right)}{K_{m\left(S\right)}}}$$

where *K*_m(S)_ and [S] are the reporter substrate’s *K*_m_ and fixed concentration used, respectively.

For irreversible inhibitors like clavulanic acid, the rate constant of inactivation, *k*_inact_, was measured directly by time-dependent inactivation of LRA-5 variants using a fixed concentration of enzyme and 100 µM nitrocefin as reporter and increasing concentrations of the inhibitor. The observed rate constant for inactivation (*k*_obs_) was determined by nonlinear least-squares fitting of the data using equation 3, as described elsewhere (42).


(3) $$A=A_{0}+v_{f}\times t+(v_{0}-v_{f})\times [1-exp(-k_{obs}\times t)]/k_{obs}$$

Then, *k*_obs_ values were plotted against the inhibitor concentration, and the inactivation constant, *k*_inact_, was obtained by nonlinear fitting of equation 4:


(4) $$K_{obs}=(k_{inact}\times [I]/(K_{M}+[I])$$

All analysis and plots were performed using GraphPad Prism version 5.03 (GraphPad Software, USA, https://www.graphpad.com/). The following extinction coefficients and wavelengths were used: nitrocefin (*δε*_482_= +15,000 M^−1^.cm^−1^), ampicillin (*δε*_235_= –820 M^−1^.cm^−1^), cephalothin (*δε*_273_= –6,300 M^−1^.cm^−1^), cefoxitin (*δε*_260_= –6,600 M^−1^.cm^−1^), ceftazidime (*δε*_260_= –9,000 M^−1^.cm^−1^), ceftriaxone (*δε*_260_= –9,400 M^−1^.cm^−1^), imipenem (*δε*_300_= –9,000 M^−1^.cm^−1^), meropenem (*δε*_300_= –6,500 M^−1^.cm^−1^), and aztreonam (*δε*_318_= –750 M^−1^.cm^−1^).

### Crystallization

Initial crystallization conditions for both proteins were screened at room temperature with a Honeybee 963 robot (Genomic Solutions, Ann Arbor, MI, USA), using 96-well Greiner 609120 sitting drop plates (Monroe, NC, USA) and the JBScreen Classic and Pentaerythritol commercial kits from Jena Bioscience (Jena, Germany). The protein samples were adjusted to approximately 7 mg/mL in pre-crystallization buffer containing 10 mM Tris, 25 mM sodium chloride (pH 8.0). Drops consisted of 350 nL protein + 350 nL precipitant. After 1 week of equilibration, several conditions showed sharp needles. In most cases, the individual successful solutions generated crystals of both proteins. Optimization was performed in 24-well hanging drop VDX plates from Hampton Research (Aliso Viejo, CA, USA), increasing the volumes to 2 µL protein + 2 µL precipitant. The best crystals (approximately 50 µm × 50 µm × 500 µm) were eventually obtained using the following solutions: (i) 1.5–1.7 M sodium formate and (ii) 13%–15% (wt/vol) PEG 20000, 0.1 M magnesium chloride, 0.1 M Tris (pH 7.9–8.8). The LRA-5/CAZ complex was obtained by overnight soaking of uncomplexed crystals in their respective mother liquor supplemented with 10 mM CAZ. Crystals were cryo-protected in either (i) 7 M sodium formate or (ii) 16% (wt/vol) PEG 20000, 19% (wt/vol) PEG 400, 0.1 M magnesium chloride, 0.1 M Tris (pH 8.5), and then cooled in liquid nitrogen in Hampton Research loops.

### X-ray data collection, processing, and structure resolution

Native diffraction data were collected at 100 K in remote mode at the Proxima-2A and I03 beamlines at the SOLEIL and Diamond synchrotrons, respectively. Indexing, integration, and reduction were performed with XDS (43) and Aimless-CCP4 (44), leaving 5% of the reflections apart for cross-validation purposes. The LRA-5 structure was solved by molecular replacement as implemented in BALBES (45), using the coordinates of the PER-1 class A β-lactamase as the search model (PDB code 1E25 [46]). The oriented model, consisting of two copies of the polypeptide, was later subjected to automatic model building with ARP/wARP (47). Further cycles of model building and refinement were performed with Coot (48) and Phenix.refine (49), respectively. The coordinates of LRA-5 were then used as a template to solve the structures of LRA-5^Y69Q/V166E^ and LRA-5/CAZ. For the latter case, the coordinates of CAZ were obtained from the PDB structure 2ZQD and the generation of the ligand restraints was performed with eLBOW (49). Clear and definite electron density for CAZ was found in the active site in each chain. All structures were subjected to validation with MolProbity (50) and with the validation module of Coot. Models were visualized with PyMOL 2.4.1 (34). Details of the data collection and refinement are shown in Table 5.

### Protein data bank deposition

The coordinates and structure factor amplitudes of wild-type LRA-5, its complex with ceftazidime, and the uncomplexed LRA-5^Y69Q/V166E^ β-lactamases were deposited under accession codes 8EO5, 8EO6, and 8EU7, respectively.

### Circular dichroism

Spectra for the wild-type LRA-5 and derived mutants were recorded on a Jasco J-810 spectropolarimeter. Data in the near UV (250–320 nm) or in the far UV (200–250 nm) regions were collected at 25°C in 10- or 1 mm path length cuvettes, respectively. A scan speed of 20 nm/min with a time constant of 1 s was used for all proteins. Each spectrum was measured at least three times, and the data were averaged to minimize noise. Molar ellipticity was calculated as described elsewhere (51).

### Enhanced sampling molecular dynamics simulations

The structures of wild type LRA-5 (PDB 8EO5) and the LRA-5 double substituted Tyr69Gln/Val166Glu (LRA-5^Y69Q/V166E^, PDB 8EO7) are those reported in this work. The missing regions in the LRA-5^Y69Q/V166E^ variant were reconstructed by extracting the corresponding segment from the LRA-5 wild-type structure using the MolSoft ICM-Pro Suite (https://www.molsoft.com/). The ICM mutagenesis program was employed for the *in silico* construction of the single variants (LRA-5^V166E^ and LRA-5^Y69Q^) from the LRA-5 wild-type structure (52). The wild-type and single substituted systems were prepared for simulations using a high-throughput molecular dynamics (HTMD) protocol (53). The protein parameters were defined using the Amberff14SB force field (54), and explicit TIP3P water molecules were included in a cubic box with its edge at least 10 Å from the closest solute atom (55). The systems were generated using 0.15 M NaCl. The electrostatic interaction distances were set to ≤ =8 Å. Long-range electrostatic interactions were calculated using the particle-mesh Ewald summation (56). The systems underwent a minimization process with 1,000 steps of steepest descent integrator, followed by a 5 ns equilibration in the NPT ensemble with a Berendsen barostat at 1 atm (57). The temperature was maintained at 300 K using a Langevin thermostat. To enhance sampling, Well-Tempered Metadynamics simulations (wt-MetaD) were performed using the equilibrated structures of both the wild-type LRA-5 and single substituted variant structures (58). These simulations were carried out using the ACEMD software and the PLUMED 1.3 plug-in (59). The *φ* and *ψ* dihedral angles of residues 69 and 166 were selected as CV1 and CV2, respectively. Gaussian distributions with widths of 0.1 radians were applied to the two collective variables (CVs) to add the bias, respectively, while the height of the Gaussians was set at 0.5 kJ/mol. The Gaussians were deposited every 4 ps, ensuring a deposition rate of 0.125 kJ/(mol·ps). The bias factor remained constant at 15. The simulations were run for 5 µs to achieve maximum convergence of the free energy landscape.

The OneOPES MD simulations were carried out with the GROMACS 2023.5 engine patched with the PLUMED 2.11 plugin (60, 61). Energy minimization was performed using the steepest descent algorithm for up to 50,000 steps, with a convergence criterion of 1,000 kJ mol⁻¹ nm⁻¹. The systems were subsequently equilibrated through a four-step protocol. Initially, heating was performed in the NVT ensemble for 20 ns using the V-rescale thermostat at 300 K, with positional restraints applied to the protein Cα atoms (force constant (kpr) = 1,000 kJ mol⁻¹ nm⁻²) (62). This was followed by equilibration in the NPT ensemble, employing the V-rescale thermostat (300 K) and the Parrinello–Rahman barostat (1 atm) (63). Three successive equilibration phases of 20, 10, and 5 ns were conducted, during which the positional restraints on the Cα atoms were progressively reduced to 1,000, 100, and 10 kJ mol⁻¹ nm⁻², respectively. The final equilibrated structure was employed as the initial configuration for the production simulations. A 5 ns unbiased production run was performed for both protein systems in the NVT ensemble with a 4 fs integration time step. The temperature was maintained at 300 K using the V-rescale thermostat. Periodic boundary conditions were imposed, and long-range electrostatic interactions were treated with the particle–mesh Ewald (PME) method (56), while a cut-off of 10.0 Å was applied for short-range interactions. The OneOPES simulations comprised eight exchanging replicas (0–7) (64), each employing four collective variables in OPES Explore (65), namely the *φ* and *ψ* backbone torsional angles of residues 69 and 166. The Gaussian widths (*σ* values) for each CV were derived from a 5 ns unbiased production run and were identical across all replicas. For the WT system, the *σ* values were 0.1490, 0.1392, 0.1140, and 0.1423 for the *φ* and *ψ* dihedral angles of V166 and Y69, respectively, while for the LRA-5^Y69Q/V166E^ double mutant were 0.1555, 0.1510, 0.1073, and 0.1271. The bias potential was set with a barrier height of 100 kJ mol⁻¹ and the frequency of bias deposition of 5,000 steps. Replicas 1–7 additionally employed OPES MultiThermal (65), with temperature ranges of 300–310 K (replica 1), 300–320 K (replica 2), 300–330 K (replica 3), 300–340 K (replica 4), 300–350 K (replica 5), 300–360 K (replica 6), and 300–370 K (replica 7). Exchanges among the eight replicas were attempted every 1,000 simulation steps. The thermostat and barostat settings followed the protocol described in the unbiased MD simulations section. The total simulation time amounted to approximately 3.6 µs for the WT system and 4.0 µs for the LRA-5^Y69Q/V166E^ double mutant.

The structural figures were generated using Protein Imager (https://3dproteinimaging.com/), a modified version of open-source PyMOL (https://github.com/bieniekmateusz/pymol-mdanalysis), and Molsoft ICM-Pro package (http://www.molsoft.com).

## SUPPLEMENTAL MATERIAL

Supplemental material aac.00675-25-s0001.docx Fig. S1 to S10.

ASM does not own the copyrights to Supplemental Material that may be linked to, or accessed through, an article. The authors have granted ASM a non-exclusive, world-wide license to publish the Supplemental Material files. Please contact the corresponding author directly for reuse.
