# Supplementary material for: Proposal of metagenomic-origin LRA-5 as a precursor of active β-lactamases through Tyr69Gln and Val166Glu amino acid substitutions: a functional and structural analysis
Source: Antimicrob Agents Chemother. 2025 Nov 28;70(1):e00675-25. doi: 10.1128/aac.00675-25 (PMC12777564; doi:10.1128/aac.00675-25)
Supplement: Supplemental material — Fig. S1 to S10. [file aac.00675-25-s0001.docx]

**Supplementary material**

**Proposal of metagenomic-origin LRA-5 as a precursor of active β-lactamases through Tyr69Gln and Val166Glu amino acid substitutions: a functional and structural analysis.**

Gabriela D’Amico González, María Margarita Rodríguez, Pedro Penzotti, Florencia Brunetti, Barbara Ghiglione, Luke A. Moe, Daniela Centrón, Gabriel Gutkind, Lin Gao, Shozeb Haider, Rachel A. Powers, Sebastián Klinke, Pablo Power

**Figure S1** – Control of unspecific cefepime hydrolysis. Orange plot shows the hydrolysis of 100 µM cefepime in presence of 1 µM LRA-5, at 260 nm. Blue plot represents the control reaction of 100 µM cefepime without addition of enzyme.


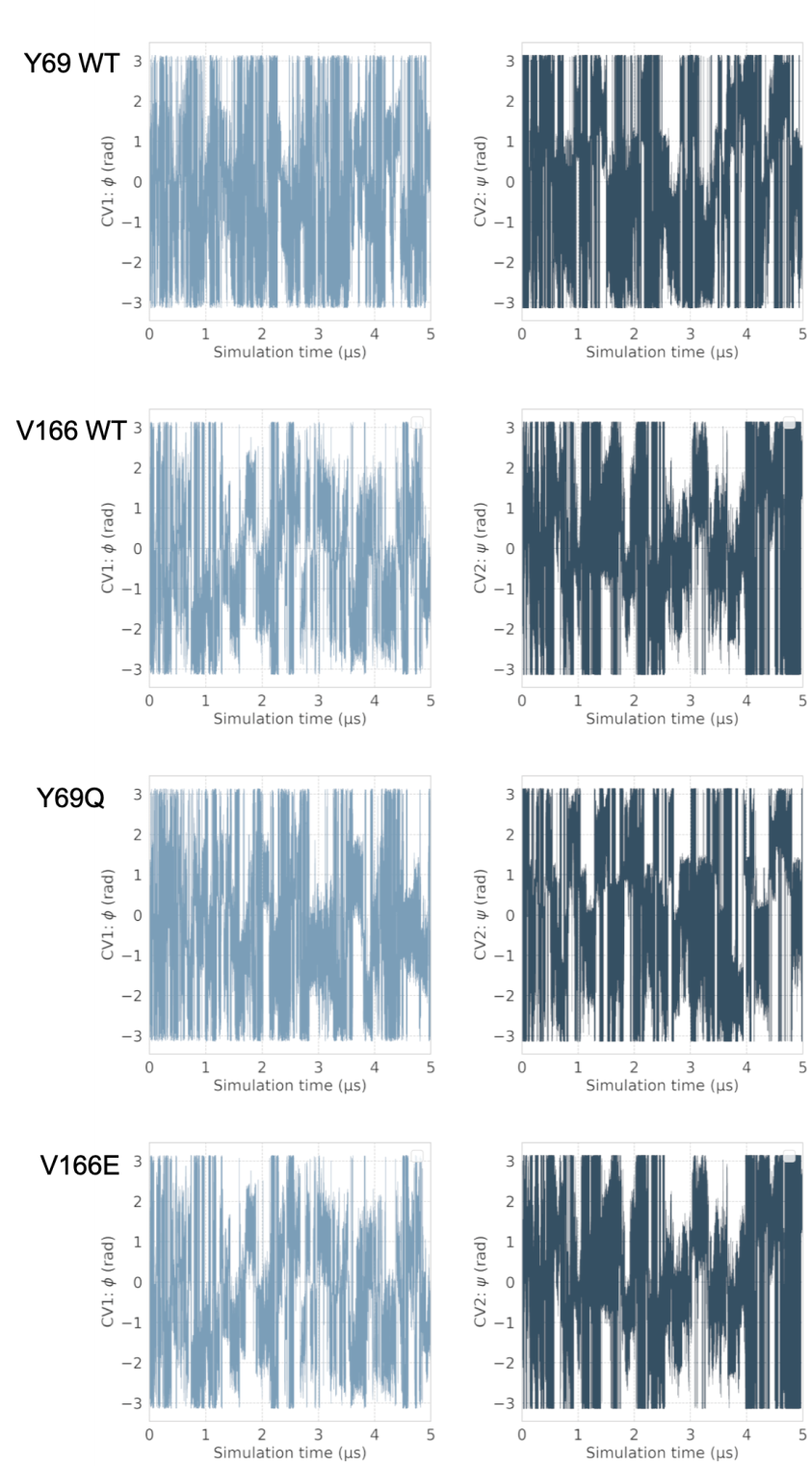


**Figure S2** - Diffusive behaviour of Y69 and V166 in the wild-type protein and in the single substituted variants along the CVs φ and ψ in well-tempered MetaD simulations.


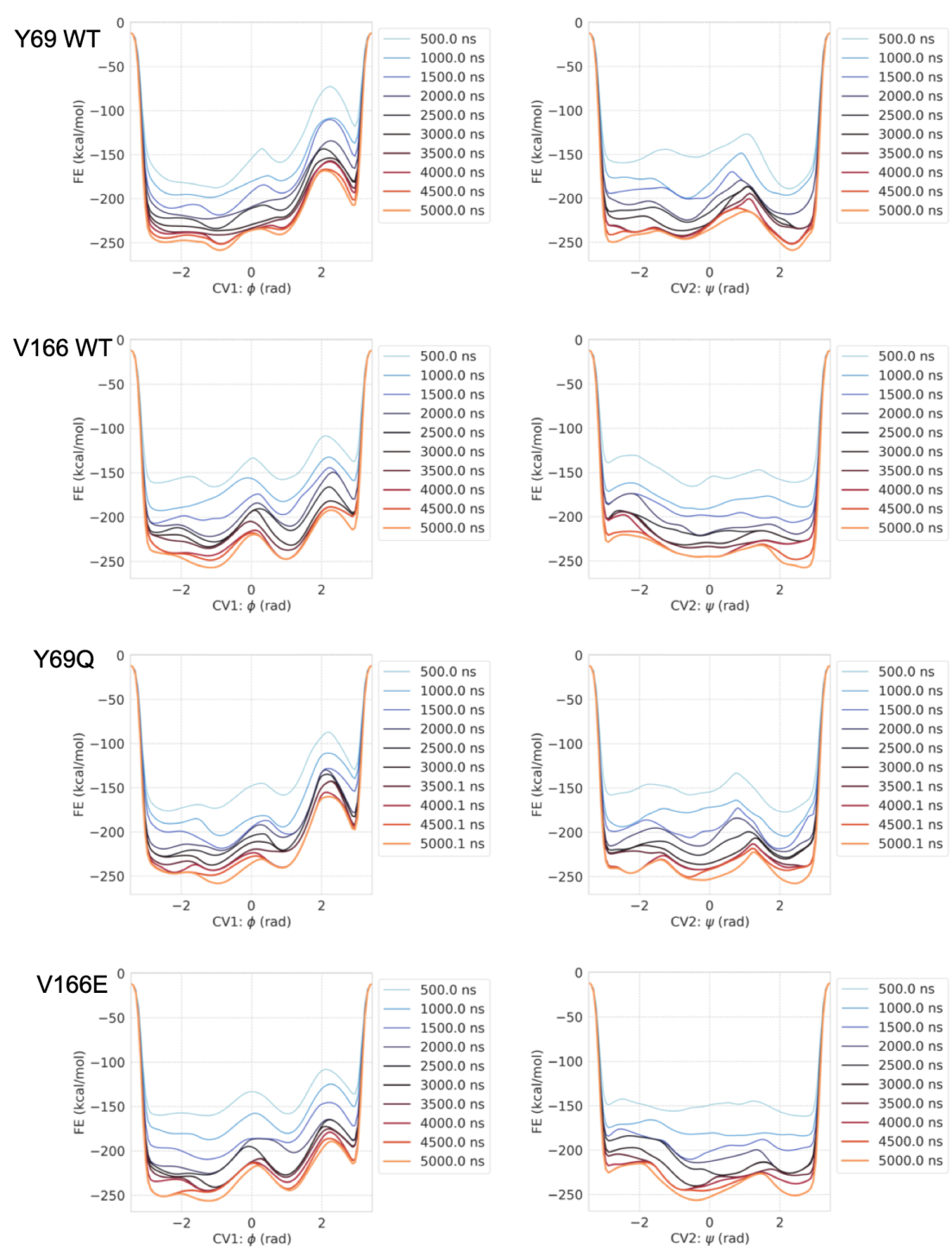


**Figure S3** - Convergence plots for CV1 φ and CV2 ψ from the wild-type protein and the single substituted variants in well-tempered MetaD simulations


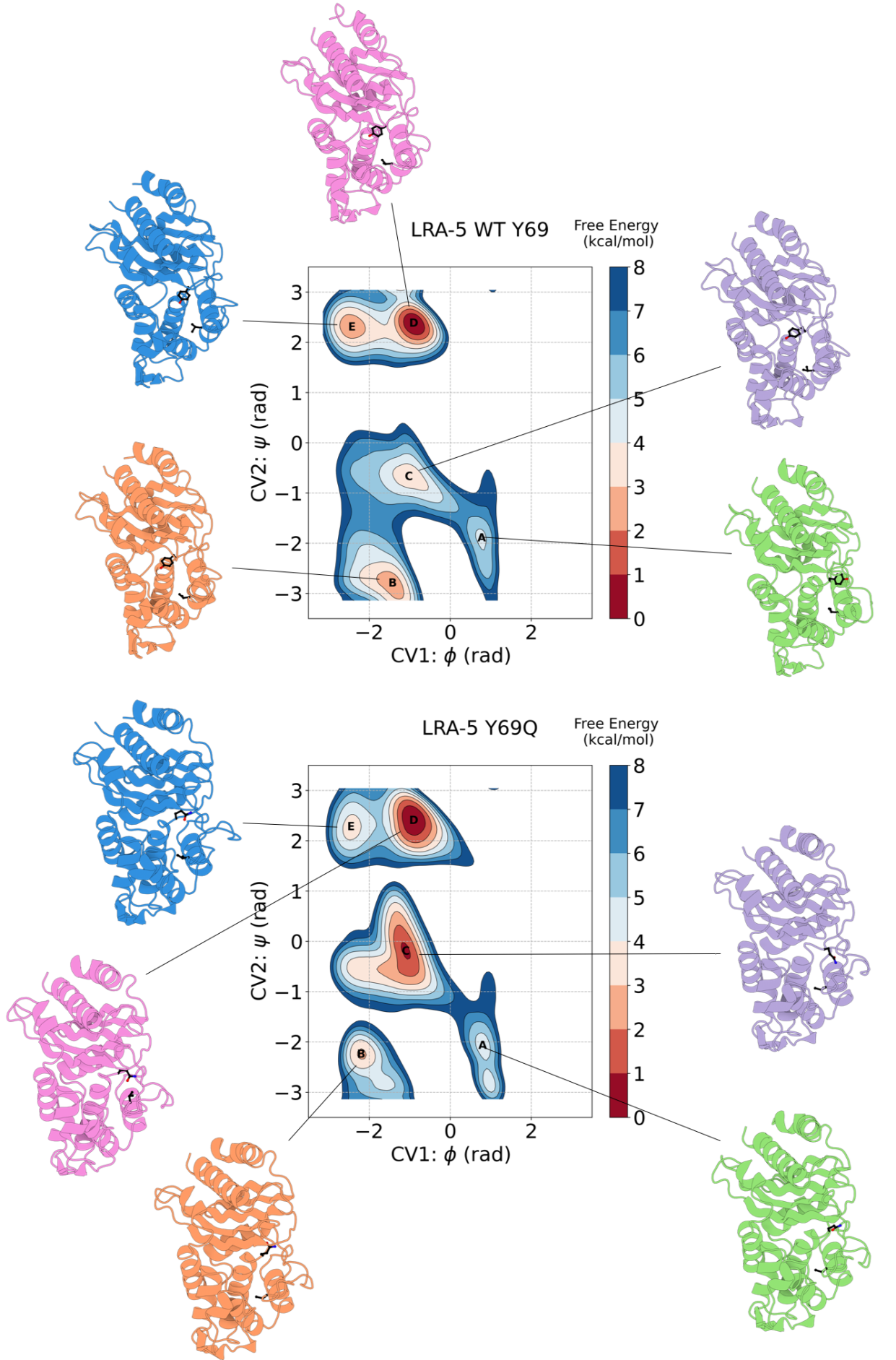


**Figure S4** – Free energy plots for wild-type LRA-5 and LRA-5^Y69Q^ as a function of φ (CV1) and ѱ (CV2) dihedral angles of residue 69. The overall topological conformations and the side chains of Y69/Q69 and V166 have been illustrated as black sticks.


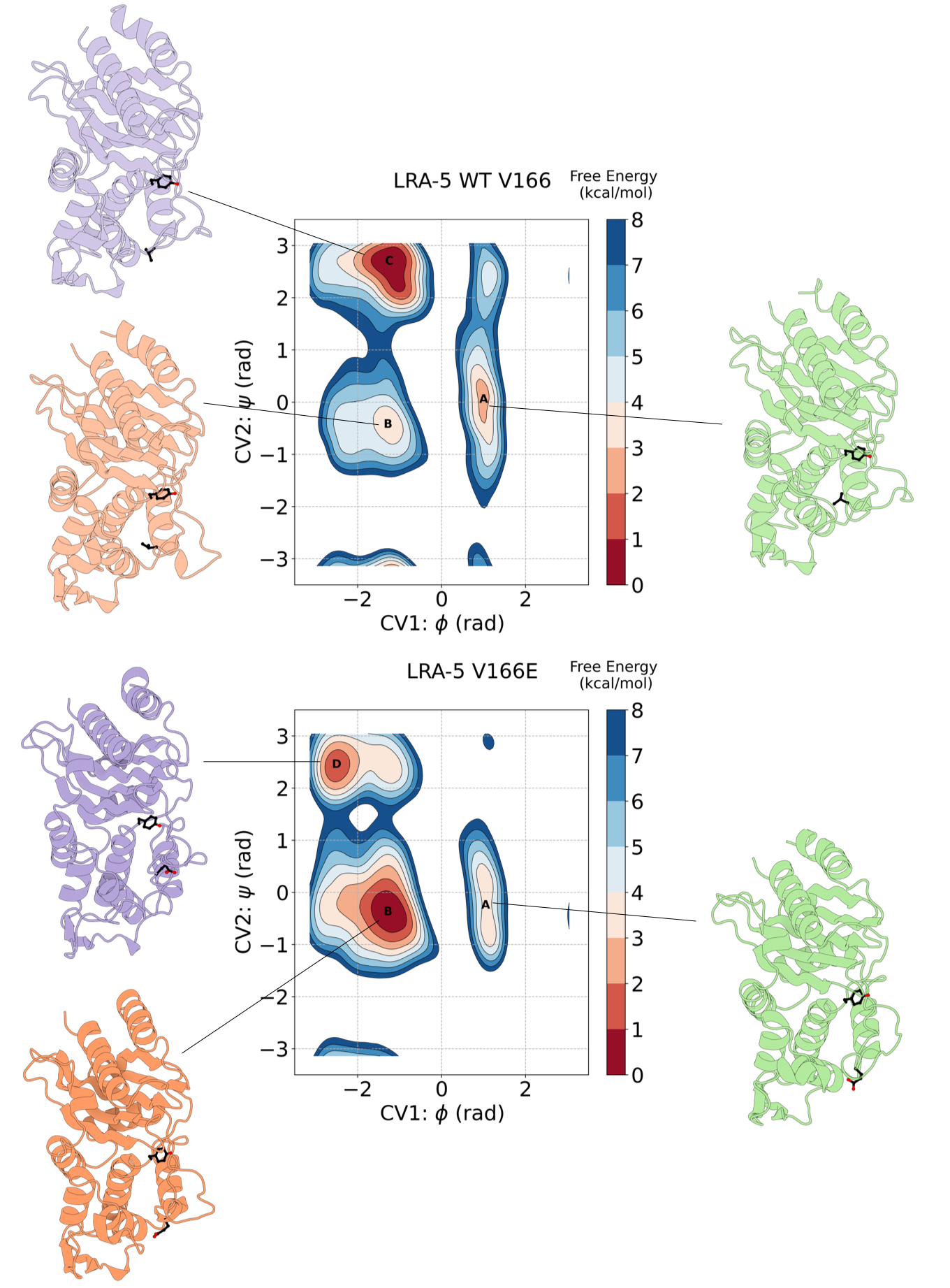


**Figure S5** – Free energy plots for wild-type LRA-5 and LRA-5^V166E^ as a function of φ (CV1) and ѱ (CV2) dihedral angles of residue 166. The overall topological conformations and the side chains of Y69 and V166/E166 have been illustrated as black sticks.


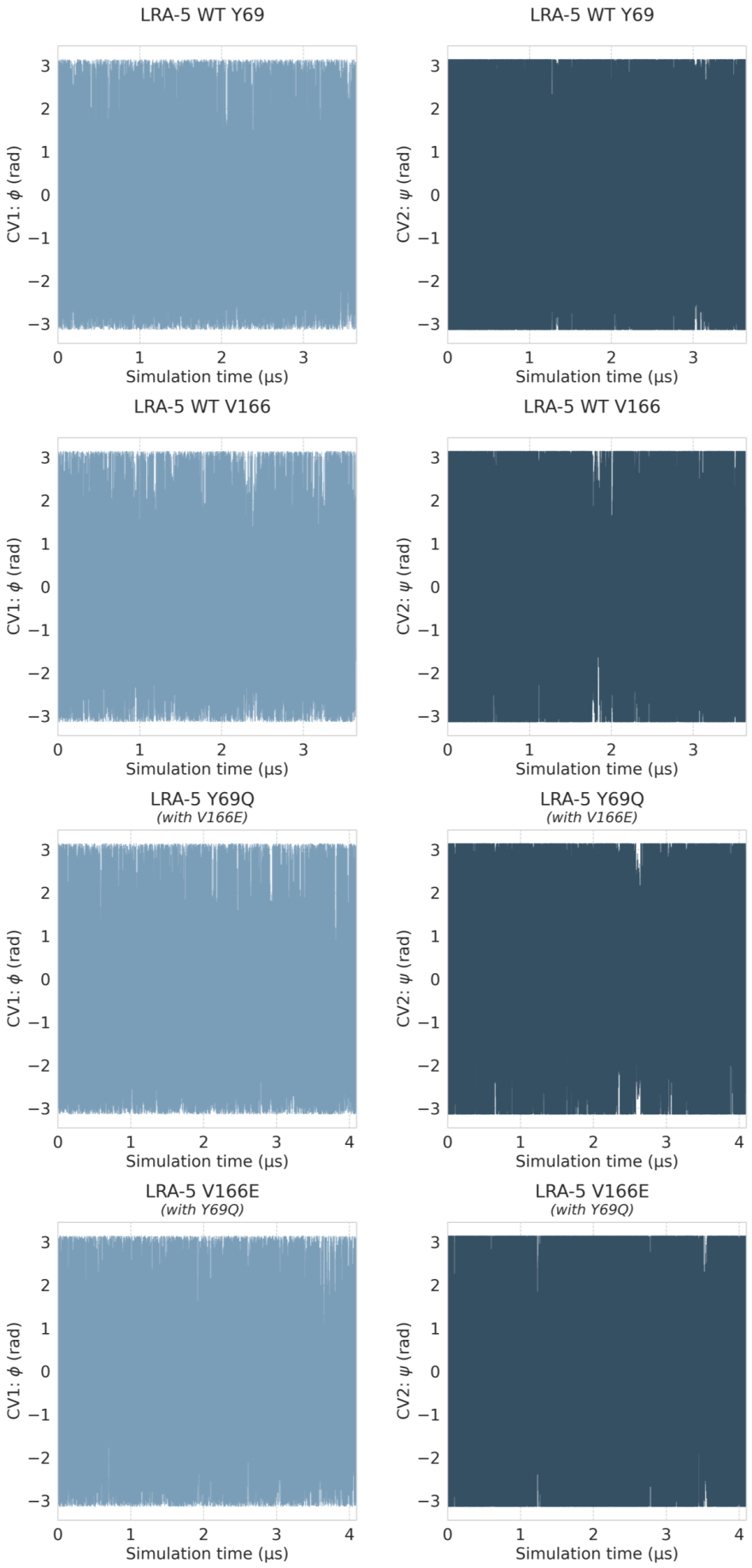


**Figure S6** - Diffusive behaviour of Y69 and V166 in the wild-type and double substituted variants along the CVs φ and ψ in OneOPES simulations


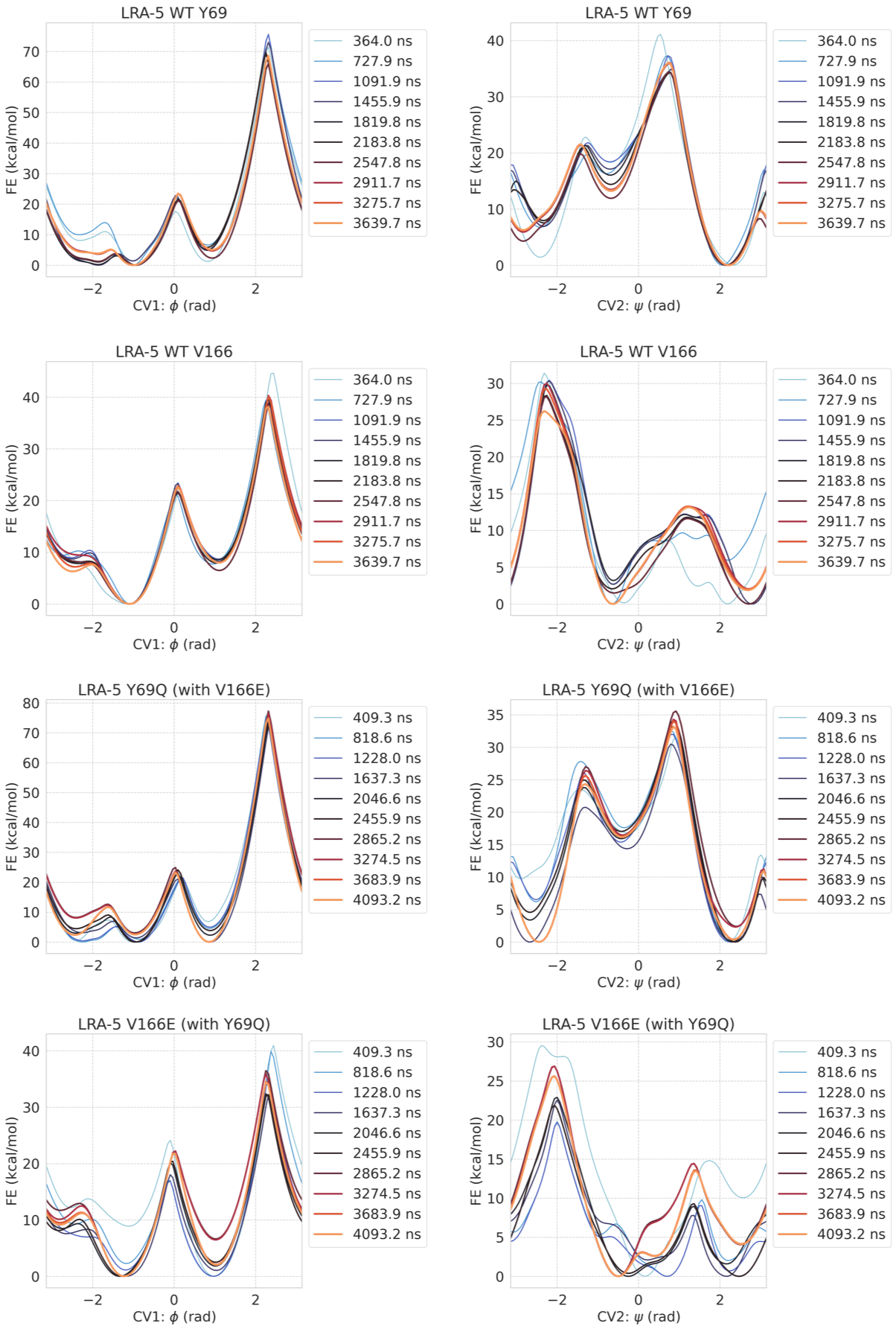


**Figure S7** - Convergence plots for CV1 φ and CV2 ψ from Y69 and V166 in the wild-type protein and double substituted variants in OneOPES simulations


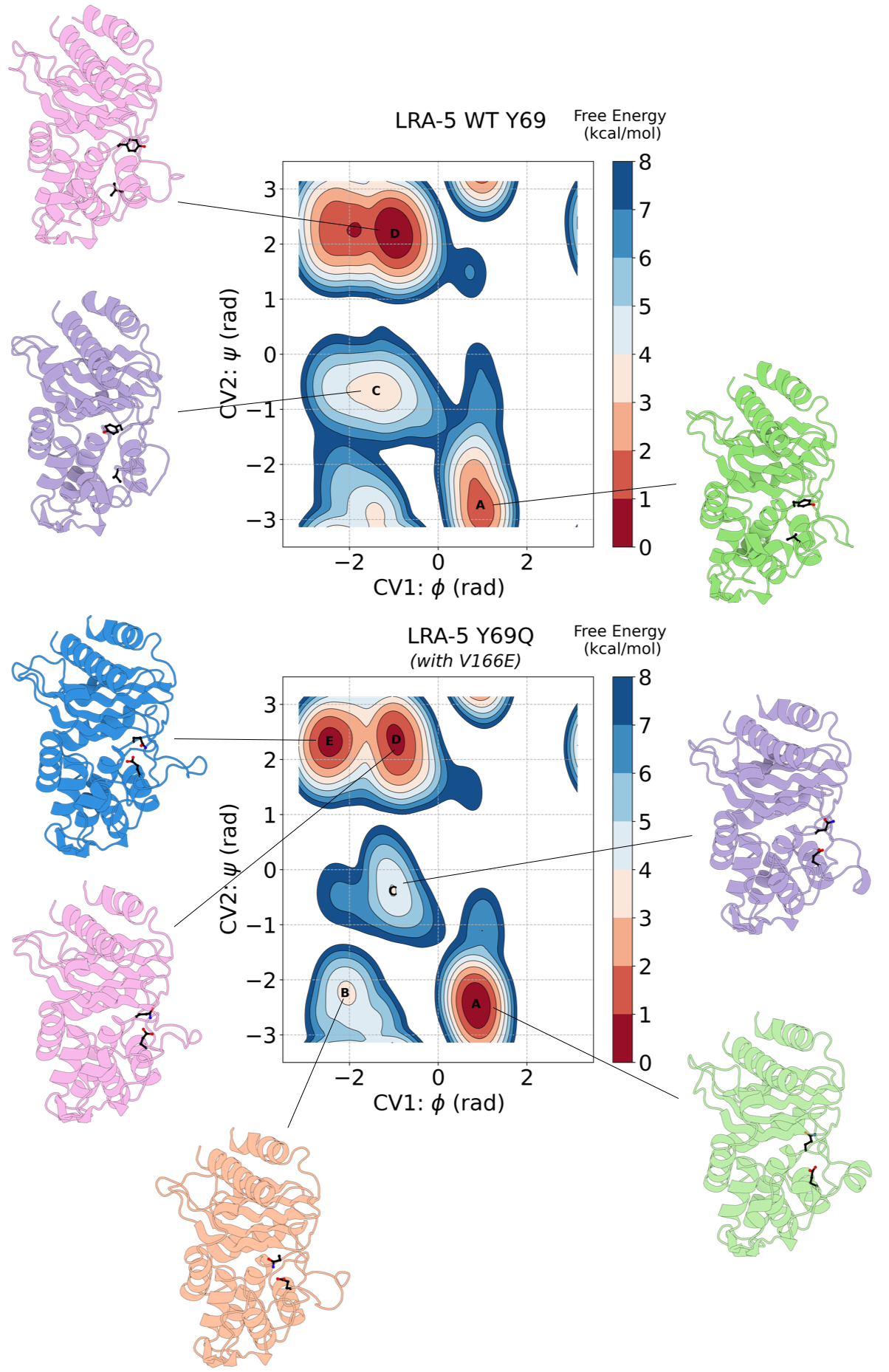


**Figure S8** – Free energy plots for wild-type LRA-5 and LRA-5^Y69Q/V166E^ as a function of φ (CV1) and ѱ (CV2) dihedral angles of residue 69. The overall topological conformations and the side chains of Y69/Q69 and V166 have been illustrated as black sticks.


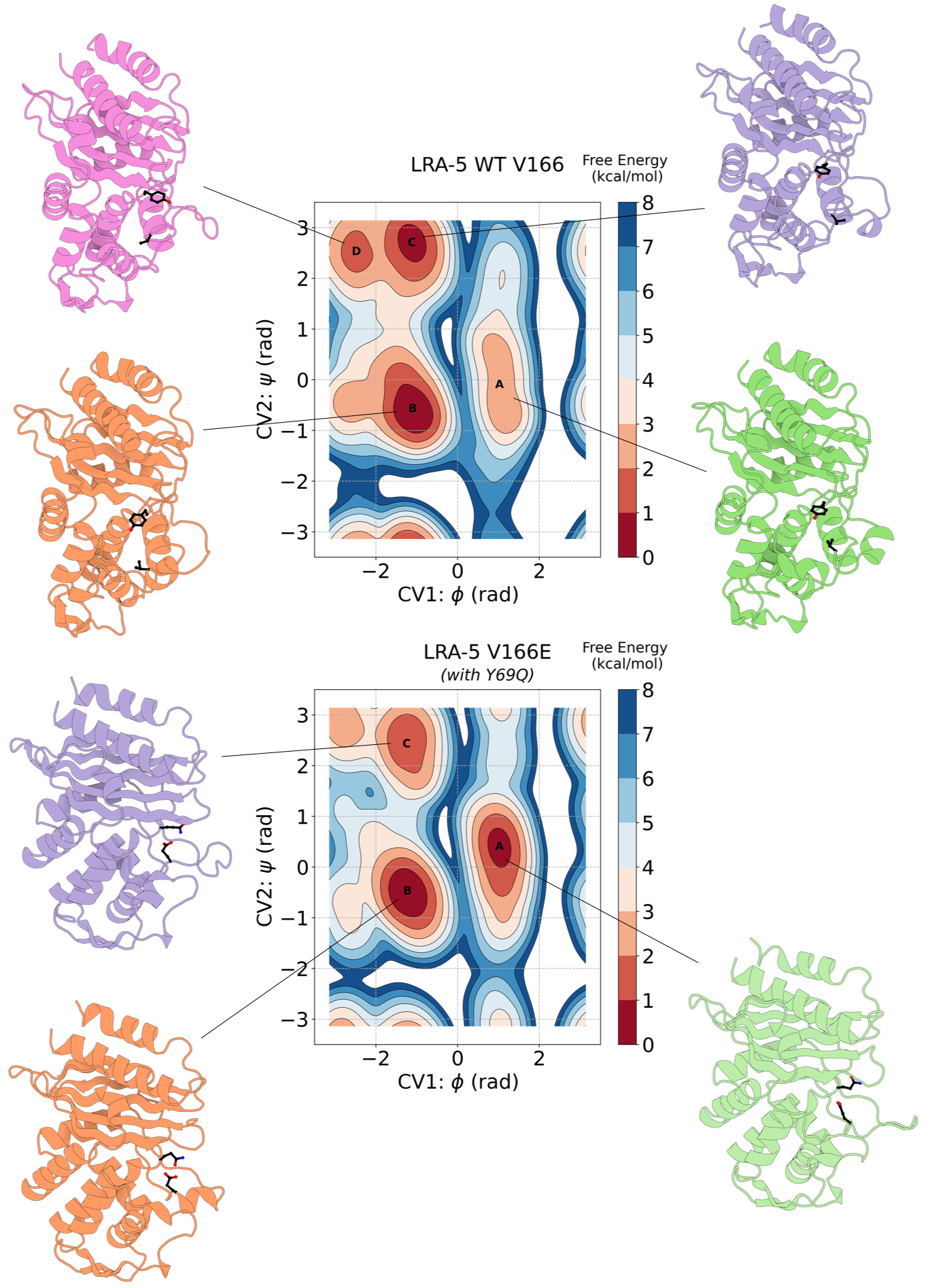


**Figure S9** – Free energy plots for wild-type LRA-5 and LRA-5^Y69Q/V166E^ as a function of φ (CV1) and ѱ (CV2) dihedral angles of residue 166. The overall topological conformations and the side chains of Y69 and V166/E166 have been illustrated as black sticks.

**
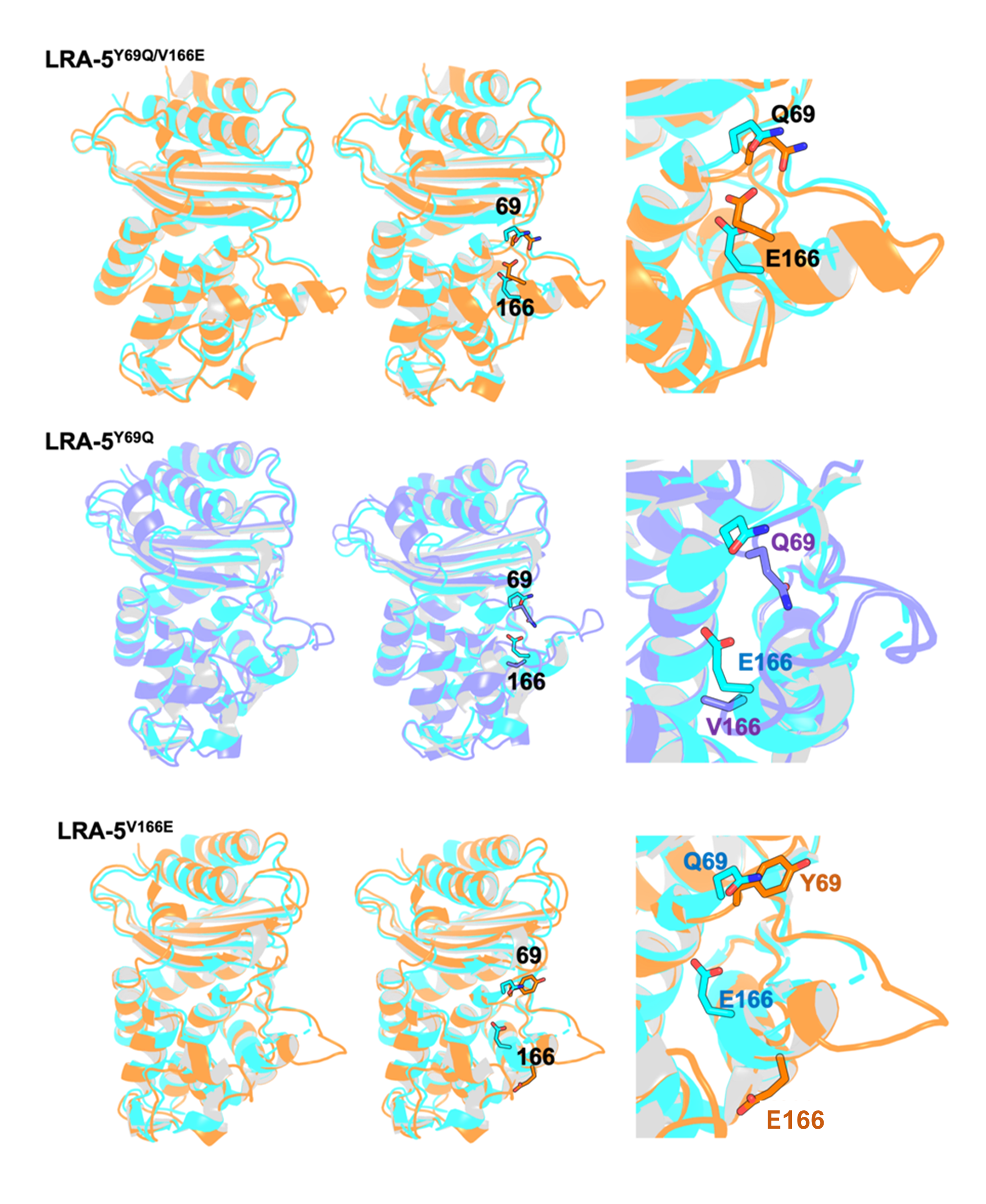
**

**Figure S10** – Superimposition of the double substitution LRA-5^Y69Q/V166E^ crystal structure (PDB 8EO7, cyan) on the representative structures extracted from basin B in double mutant (top row, orange); basin C in the single substituted LRA-5^Y69Q^ (middle row, purple) and basin B in the single substituted LRA-5^V166E^ (bottom row, orange) simulations. The spatial positions of residues 69 and 166 are labelled. In the double mutant, the overall topology and spatial positioning of Q69 and E166 align well between the crystal and the simulated structure. In the single mutants, the substituted residues are unable to match the crystallographic conformation.
